# Exposure to brominated flame retardants *in utero* and through lactation delays the development of DMBA-induced mammary cancer: potential effects on subtypes?

**DOI:** 10.3389/fendo.2024.1429142

**Published:** 2024-11-14

**Authors:** Melany N. Juarez, Alec McDermott, Michael G. Wade, Isabelle Plante

**Affiliations:** ^1^ INRS-Centre Armand-Frappier Santé Biotechnologie, Laval, QC, Canada; ^2^ Environmental Health Science and Research Bureau, Healthy Environments and Consumer Safety Branch, Health Canada, Ottawa, ON, Canada

**Keywords:** mammary gland, brominated flame retardants, cancer, 7,12-dimethylbenz[a]anthracene (DMBA), gestational-lactational exposure, endocrine disrupting compounds (EDCs)

## Abstract

**Introduction:**

Brominated flame retardants (BFRs) are chemical compounds used to reduce the flammability of various products; some BFRs exhibit endocrine-disrupting properties and can leach into the environment leading to human and wildlife exposure. The mammary gland has specific vulnerability windows during which it is more sensitive to the effects of endocrine disrupting compounds (EDCs), such as the *in utero* life, puberty and pregnancy. Our previous studies revealed precocious mammary gland development, disruptions in junctional proteins, and altered proliferation-apoptosis balance during puberty in rats exposed to BFRs *in utero* and through lactation. Such effects have been associated with increased mammary cancer risk.

**Objective:**

The current study aimed to determine if *in utero* and lactational exposure to BFRs renders the mammary gland more susceptible to 7,12-dimethylbenz[a]anthracene (DMBA)-induced mammary cancer.

**Methods:**

Dams were exposed to a BFRs mixture (0. 0.06 or 60 mg/kg/day), and mammary cancer was induced in pups using DMBA at post-natal day 46. Tumors onset and growth were monitored, and tumors were characterized using histology and molecular biology.

**Results:**

Although BFRs exposure did not significantly affect mammary tumor number or burden, it showed significant delay in mammary tumor onset and growth in BFR-exposed animal. These effects could potentially be due to BFRs’ impact on cellular responses, DMBA metabolism, or mammary gland shift of the sensitivity window. Molecular analysis of mammary tumors showed a shift in the ratio of luminal A, luminal B, and (HER2)-enriched tumors, and an increase in triple-negative breast cancer (TNBC) subtypes in BFR-exposed animals. Additionally, BFRs exposure showed lung lesions indicative of inflammation, independent of mammary cancer development.

**Conclusion:**

Our study highlights the complex relationship between BFRs exposure and mammary cancer risk, emphasizing the need for further investigation into underlying mechanisms and long-term effects of BFRs on mammary gland development and carcinogenesis.

## 1 Introduction

Brominated flame retardants (BFRs) are chemicals which inhibit the ignition and spread of flames. They were routinely added to several synthetic materials to meet fire safety standards (1, 2). Most BFRs are not covalently linked with the polymer matrices to which they are added and tend to leach into the environment. They have been detected in house dust, sediments, sludge, air, soil and water, as well as in human and wildlife tissues in the United States, Canada, Europe and Asia (3–7). Polybrominated diphenyl ethers (PBDEs) and hexabromocyclododecane (HBCDD) were some of the most extensively used BFRs, and they were added to commercial goods in the form of commercial mixtures (8, 9). As a result of the evidence of persistence and bioaccumulation, the European Union restricted the sale of Penta- and Octa-BDEs in 2005 and added some congeners to this regulation in 2019 (1, 10). In addition, the trade, import, production and use of PBDEs and HBCDDs have been restricted under the Stockholm Convention, but are not necessarily banned in all the signatories. BFRs can still be found in waste and recycled materials, they are still added to some electrical goods, and used in the aviation and automobile industry (11, 12), thus resulting in chronic environmental and human exposure.

Human exposure mainly results from BFRs ingestion and inhalation of dust (7, 13). Several studies have found BFRs in indoor dust, where the degree of degradation and dispersal are considerably reduced compared with other matrices exposed to sunlight and other environmental forces (7, 14–16). Indoor concentrations of PBDEs in the USA range from 700 to 30,100 ng/g of dust, where BDE-209 was found at the highest concentrations and represented in 88% of the samples (7). The ingestion of contaminated fish, meat, eggs and dairy products is another source of human exposure (17, 18). Toddlers are the population with the greatest exposure due to constant contact with indoor dust, toy-leaching BFRs, hand-to-mouth behavior, diet and consumption of breast milk (19, 20).

The association between exposure to PBDEs and disruption in reproductive development, neurobehavior, thyroid hormone physiology, as well as male and female reproductive function has been the subject of many reviews (21–27). The mechanism(s) underlying BFR-induced health effects remain to be fully elucidated but it is clear that PBDEs or their metabolites interfere with several hormonal pathways including thyroid (21, 28, 29), estrogen (30, 31) and androgen (32). PBDEs have a strong structural similarity with thyroid hormone, and HBCDDs can bind to thyroid hormone receptors and thyroid transport proteins with varying affinities (33). They can cause hypertrophy of the thyroid gland epithelium as well as induce hepatic cytochrome activity (28). PBDEs can bind to estrogen receptors and cause the expression or inhibition of genes which are regulated by estrogen depending on the congener (34, 35). For example, PBDEs have been shown to enhance the estradiol-mediated regrowth of the mammary glands with TEBs-like structures in a menopausal mice model, suggesting that PBDEs may increase the risk for mammary cancer development due to the activation of growth of luminal cells by estrogen-responsive mechanisms (31). Additionally, PBDEs in the presence of estrogens may affect immune modulators and the reorganization of the epithelium (31).

The development of the mammary gland occurs mainly after birth in a multistage remodeling process (36). At birth, the mammary gland is present as a rudimentary ductal structure. At puberty, in response mainly to estrogens and progesterone, the branching morphogenesis initiates, causing the ductal tree to elongate into the fat pad. This process is led by highly proliferative multilayered terminal end buds (TEBs) (37). Upon pregnancy, alveoli are generated under the combined action of progesterone and prolactin, which will secrete milk during lactation in response to the presence of oxytocin (38). At weaning, in response to the lack of demand of milk, the process of involution takes place and the mammary gland is remodeled back into its pre-pregnancy-like state (39).

There is evidence supporting that exposure to EDCs during sensitive periods of life is linked to developmental defects in the mammary glands and mammary cancer later in life as reviewed by (40, 41). These sensitive periods correlate with cellular proliferation and differentiation, and are highly regulated in response to hormones in endocrine-sensitive tissue; in the case of the mammary gland, this includes perinatal life, puberty, pregnancy and lactation (42). The influence of PBDEs on mammary gland tumors was suggested by a study showing that the majority of PBDE congeners were significantly and markedly elevated in adipose tissue of breast cancer cases compared to controls (43). Further, PBDE was shown to enhance estrogen-driven mammary gland remodeling and molecular changes suggestive of a PBDE-induced increase in duct luminal cell maturation (31). Recently, we showed that dams exposed during pregnancy and lactational to an environmentally relevant mixture of BFRs, have reduced serum levels of T4 and altered adherens junctions in the mammary glands at weaning (44, 45). Ovarian folliculogenesis and steroidogenesis were also affected (46). In pups exposed during gestation and through maternal milk, skeletal and digital abnormalities were observed (47), as well as neurodevelopment and lipid metabolism defects (48), and early onset of puberty and abnormal ovarian follicles at post-natal day 46 (PND46) (49). In addition, we have shown that pups exposed to a mixture of BFRs at 0.06mg/kg/day *in utero* and through lactation showed down-regulation of adherens junctional proteins (E-cadherin and β-catenin), gap junctional protein (p-Cx43), THR-α1 and cleaved caspase-3, suggesting a disruption in cell-cell interactions and of the balance between proliferation and apoptosis in the mammary gland at PND46 (peripubertal stage) (50). A precocious mammary gland development has also been observed in pups at PND21 (51).

Dysregulation of adherens junctions and precocious development of the breast have been associated with abnormal mammary gland development and mammary tumors for both women and animal models (41, 52–56). Based on these results, the current study thus aimed to determine whether exposure to BFRs *in utero* and through lactation could enhance the progression of DMBA-induced mammary gland carcinogenesis in exposed female pups.

## 2 Materials and methods

### 2.1 Preparation of BFRs mixture

An environmentally relevant mixture of BFRs, formulated to mimic concentrations found in Boston house dust was used (9, 57). The formulation and composition of this mixture have been described in detail elsewhere (28); briefly, three different technical PBDE mixtures (DE-71, DE-79 and BDE-209; **Supplementary Table 1**) and one HBCDD mixture were combined to give relative levels of congeners similar to that observed in house dust. The BFRs mixture was incorporated into the diet (Teklad Global 2019 diet; Envigo Laboratories, Madison, WI, USA) as described previously (44, 50, 51) at a concentration of 0, 0.75, or 750 mg of BFRs/kg to deliver a nominal daily dose of 0, 0.06, or 60 mg/kg of body weight/day, respectively. The control corresponded to the same pelleted diet without the presence of BFRs. The lowest dose approximates the maximal human exposure, calculated based on the dust ingestion rate (100 mg/day) in children (16.5 kg body weight) and then scaled human to rat body surface area ratio (1:6.9). In our previous studies, the lowest dose was associated with molecular changes observed in mammary glands of exposed pups at multiple ages (44, 50, 51), while the highest dose induced liver enlargement and enzyme induction (45). An intermediate dose was used previously in our studies but not kept in this protocol as there were no significant effects on the mammary gland development (44, 50, 51). Pelleted diets were stored at 4°C for no more than 1 month prior to feeding. Previous studies have confirmed the relative exposure levels for this type of protocol (45, 47, 58).

### 2.2 Animal exposure

The studies involving animals were conducted under the procedures provided by the Canadian Council on Animal Care. The protocol (1909–02) was reviewed and approved by the Institutional Committee for Animal Protection of the Laboratoire National de Biologie Expérimentale (LNBE), the animal facilities based at the Institut National de Recherche Scientifique (INRS).

Virgin Sprague-Dawley female rats aged 6-7 weeks were obtained from Charles River Laboratories (Charles River, St-Constant, QC, Canada) and acclimatized for 1 week on the control diet (**Supplementary Figure 1**). The estrus cycle was followed through vaginal impedance (59); an impedance higher than 3 kilo ohms was considered as an indicator of the proestrus phase, which was later confirmed with a vaginal smear before mating. Animals properly cycling were randomly divided (n ≥ 25) into the three treatments; 0, 0.75 or 750 mg of BFRs/kg and exposed through the diet for a minimum of one week before mating. Female rats in proestrus were housed overnight with a male Sprague-Dawley rat with an *ad libitum* control diet and water. Sperm-positive vaginal smears the following morning indicated mating and were considered gestational day 0 (GD0). All mated females were housed individually and allowed to deliver; the day of birth was considered post-natal day 0 (PND0). The exposure to BFRs was extended during lactation and ended at PND21 (weaning), resulting in gestational-lactational exposure for the pups (**Supplementary Figure 1**). After birth, dams’ and pups’ weights were recorded twice a week. All animals were kept under controlled light conditions (12L:12D). At PND0, litter size and stillborn pups were counted. The sex ratio was recorded at PND4, and litter size was normalized to 8 pups per litter. A ratio of 6 female/2 male was preferred when possible.

Pups were weaned at PND21 and subsequently fed the control diet. Mammary gland tumorigenesis was induced in juvenile female pups (PND46), an age when the mammary gland is highly sensitive to carcinogens, by treatment with the well-characterized mammary gland carcinogen 7,12-dimethylbenz[a]anthracene (DMBA; CASNo.:57-97-6, purity ≥95%, Sigma-Aldrich, St-Louis, MO, USA) (60). Two female pups from each litter were separated into oil- or DMBA-exposed groups. Female pups were administered corn oil or DMBA (20 mg) diluted in corn oil by gavage (administered as 20 mg/ml per animal) at PND46. Thus, one offspring per litter was considered as an observation. At the end of the protocol a total of 6 experimental groups were analyzed, composed of pups exposed through gestation and lactation to BFRs (low or high dose) or to the control diel, and later exposed to either oil- or DMBA at PND46. Animals were monitored bi-weekly for body weight and mammary gland tumor onset by palpation, and to detect any changes in general health.

### 2.3 Evaluation of tumorigenesis

Tumor onset and growth were evaluated by palpation and using a caliper bi-weekly. To do so, pups were restrained using one hand, and the presence of tumors was evaluated by palpating the region around each nipple by gently rolling the skin between the index and the thumb. Tumor onset was determined after a feeling of a mass in the mammary gland that grew from one palpation to another. The endpoint was determined when a tumor reached 1.5 cm^2^, or if a rat showed signs of critical illness (animals euthanized because of critical illness were removed from the study). Control animals, without tumors, were sacrificed at a similar time point. Female pups were euthanized by exsanguination under isoflurane anesthesia. At the time of sacrifice, the mammary gland and lungs, the prime site for mammary gland metastasis, were collected. Tumors that were identifiable by palpation and that were bigger than 1 mm^3^ were counted. The largest tumor was dissected and cut in half; one-half of the tumor was snap-frozen for protein extraction while the other half was fixed in formaldehyde 4% and later embedded in paraffin. The remaining tumors and mammary gland tissue (including other mammary glands) were fixed in formaldehyde 4% and later embedded in paraffin.

### 2.4 Histology

The mammary gland sections (5 μm) were stained using Masson’s trichrome. Briefly, paraffin sections were rehydrated and washed in distilled water. Slides were sequentially stained with Weigert’s iron hematoxylin (10 min), Biebrich scarlet-acid fuchsin (15 min), phosphomolybdic-phosphotungstic acid (20 min) and aniline blue (5 min). Between every coloration, they were washed with water. Finally, tissue sections were treated with 1% acetic acid (5 min), dehydrated and coverslipped using Permount (Fisher Scientific, Nepean, ON, Canada). Mammary gland sections were examined for the presence of hyperplasia, small tumors or other abnormalities. Mammary gland tumors were imaged with the confocal microscope (Nikon A1R+, Tokyo, Japan) and they were characterized blindly with the help of a pathologist.

### 2.5 Evaluation of metastasis

Lung metastasis was evaluated as described (61). Briefly, lungs were sampled at the time of sacrifice, inflated and fixed by injecting 10% buffered formalin through the trachea. Inflated lungs were kept in buffered formalin for at least 48 h, and visible lesions present on the lung surface were counted (macrotumors). Lungs were embedded in paraffin and cut using a microtome (5 μm). Sections were stained using hematoxylin and eosin for microscopic lesions detection (microtumors) and examined by a pathologist. To confirm the mammary gland origin of observed lesions, sections were stained for mammaglobin as previously described (61). Briefly, rehydrated sections were probed using anti-mammaglobin (PA5104457, Thermo Scientific Pierce, Ottawa, ON, Canada), and visualized using Vectastain ABC-HRP kit (Vector laboratories, Newark, CA, USA) and counterstained with hematoxylin (SL100 Vintage Hematoxylin, StatLab, McKinney, TX, USA). As previously described, imaging was performed by the confocal microscope (Nikon A1R+). At least 5 random pictures were taken per slide, per lobe and analyzed blindly.

### 2.6 Protein analysis

Tissues were snap frozen, limiting the action of proteases, and stored at -80°C. Protein extraction was performed by crushing the tissues into powder on dry ice and homogenizing in cold Triple-detergent buffer (50 mM Tris,150 mM NaCl, 0.02% sodium azide, 0.1% SDS, 1% Nonidet P40, 0.5% sodium deoxycholate, pH: 8) supplemented with sodium fluoride 1.25 M, sodium orthovanadate 1M and a cocktail of inhibitors 1x (Halt Protease and phosphatase cocktail inhibitor, Fisher Scientific Canada, Nepean, ON, Canada) (50). Samples were homogenized to ensure cell lysis. After sonication, samples were centrifuged (13000 x g for 10 min at 4°C) and the supernatant was stored at -80°C.

Quantification of total protein was performed by using a bicinchoninic acid (BCA) protein assay reagent kit (Thermo Scientific). Protein samples were resolved on stain-free acrylamide gels (TGX Stain-Free FastCast Acrylamide kit, 10%, Bio-Rad, Mississauga, ON, Canada) and transferred onto polyvinylidene fluoride (PVDF) membranes using the Trans-Blot Turbo Transfer System (Bio-Rad). After transfer, total lane proteins were visualized using the ChemiDoc MP imaging system (Bio-Rad) and used for normalization. Membranes were then blocked with TBS-tween 0.1% + 5% dry milk or 3% BSA and probed to primary antibodies: E-cadherin, β-catenin, keratin 14 (K14), keratin 18 (K18), connexin 43 (Cx43), estrogen receptors (ERβ, ERα), progesterone receptors (PRA, PRB), proliferating cell nuclear antigen (PCNA) and human epithermal growth factor receptor 2 (HER2; also referred as ErbB2 or HER2/neu) and horseradish peroxidase (HRP)-conjugated secondary antibodies (**Supplementary Table 2**). The expression of proteins was quantified in density, normalized to the total proteins in each lane using the software Image Lab (Bio-Rad) and compared to a pool of proteins of all the samples included in every gel (62).

### 2.7 Statistical analysis

The data was analyzed using GraphPad Prism 8 (https://www.graphpad.com) or RStudio (https://posit.co). First, the presence of outliers was determined with the ROUT test. The normal distribution of each data set was assessed with the D**’**Agostino & Pearson omnibus normality test and Shapiro-Wilk normality test. Data that satisfied assumptions of normality and homoscedasticity were analyzed using one-way ANOVA followed by Tukey**’**s multiple comparison tests. Otherwise, a Kruskal-Wallis test followed by Dunn**’**s multiple comparison test was used. For all experiments and statistical analyses, 1 pup per litter was evaluated.

## 3 Results

### 3.1 Gestational and lactational exposure to BFRs does not affect the general health of pups

We successfully mated n ≥ 10 dams per treatment. There was no significant difference in the number of successful pregnancies per mating, pups per successful pregnancy, the female vs. male ratio of pups, the general health, or the body weight of both dams and pups throughout the experiment (**Supplementary Figure 2**). Nevertheless, we noticed a higher proportion of stillborn pups from dams on either BFRs diets in comparison to the control, but this effect failed to reach significance (p = 0.4806 for control vs low dose and p = 0.1024 control vs high dose) (**Supplementary Figure 2**). These results confirm the lack of toxicity for pregnancy outcomes for these treatments as previously observed (47). Male pups were euthanized at PND21 and their mammary glands were harvested to assess the density and area of the epithelium. No changes were detected between the BFRs exposed animals and the control (data not shown).

### 3.2 BFRs-exposure rats resulted in a delayed onset of mammary tumor

**Figure 1:**
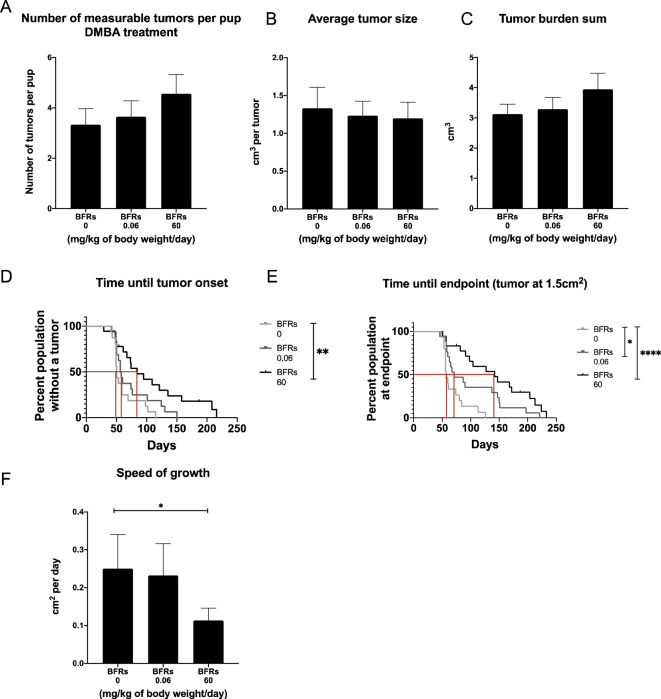
Effects on tumor onset and measurements after an exposure *in utero* and through lactation to a brominated flame retardant (BFR) mixture and a gavage with DMB at PND46. No significant differences were observed for number of tumors **(A)**, average tumor size **(B)** and tumor burden sum (considered as the sum of the estimated volume of all tumors per animal) **(C)** between treatments (0, 0.06 and 60 mg/kg of body weight/day). There was significant delay in tumor onset for the pups exposed to the high dose of BFRs, as measured by the number of days between DMBA exposure and the first tumor palpation (p=0.0039) **(D)**. A delay was observed in survival in BFR-treated group, as defined by the time between DMBA- exposure (PND46) and the endpoint for the animal (tumor reaching 1.5 cm²) **(E)**. The median survival was identified at 57 days for 0 mg/kg of body weight/day, 71 for 0.06 mg/kg of body weight/day and 142 for 60 mg/kg of body weight/day (p=0.0133 control vs low dose and p < 0.0001 control vs high dose) **(E)**. Additionally, there was a significant decrease in the speed of tumor growth (from tumor onset until the endpoint, cm² per day) for the high dose (60 mg/kg/day) when compared to the control diet (p = 0.0014) **(F)**. p-values were calculated using Mantel-cox test, Kruskal-Wallis statistical test or ANOVA. (n ≥ 10 pups, 1 per litter).

Only animals who received DMBA developed mammary tumors, meaning that BFRs by themselves do not induce the development of mammary cancer after *in utero* and lactational exposure at the time span given for this protocol. Surprisingly, within the DMBA-treated pups, exposure to BFR resulted in a dose-related delay in the time from DMBA treatment to the palpation of the first tumor (**Figure 1E**) although this delay only reached significance for the high dose. The median time after gavage until the first palpated tumor was 50 days for the control group but 57 and 84 days after the gavage for the low and high doses, respectively. The median survival from gavage and until endpoint (1.5 cm^2^) was 57 days for control, 71 for the low dose and 142 days for the high dose (**Figure 1F**). Additionally, the average speed of growth was decreased for the BFR-exposed animals (**Figure 1D**), showing a statistically decreased cm^2^ per day for the high dose when compared to the control. Measurable tumors appeared to be more numerous in BRFs-treated animals, as an average of 3.3 ± 0.6 measurable tumors were counted at the time of sacrifice for the control group, compared to 3.6 ± 0.6 and 4.5 ± 0.8 tumors for the low and the highest doses (**Figure 1A**) but this difference failed to reach statistical significance (p > 0.05). Similarly, the total tumor burden was defined as the sum of the estimated volume of all tumors per animal, which was slightly higher in the animals exposed to BFRs, while the average tumor size for the three groups does not show any difference (**Figures 1B, C**). These results suggested that animals pre-exposed to BFRs tend to develop more DMBA-induced tumors that develop later and grow slower than control animals.

### 3.3 The BFRs treatments induce changes in markers of mammary cancer in DMBA-induced tumors

**Figure 2:**
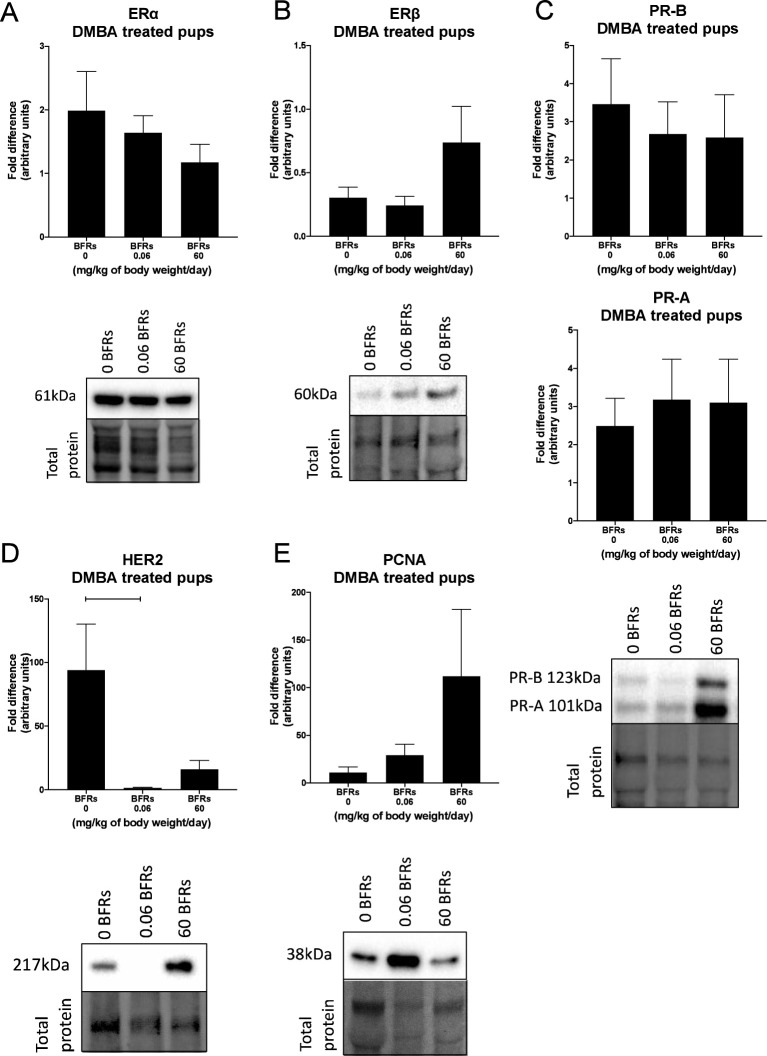
Effects of exposure *in utero* and through lactation to a brominated flame retardant (BFR) mixture on protein levels of cancer-related markers in DMBA-induced cancer in female pups. Semi-quantitative Western Blot analysis of total proteins extracted from the mammary tumors after indirect exposure to 0, 0.06 or 60 mg/kg of body weight/day and gavage by DMBA at PND46. Graphs show average expression of Estrogen Receptor alpha (ERα) **(A)**, beta (ERβ) **(B)**, Progesterone Receptors isoforms A and B (PR- A, PR-B) **(C)**, Human epidermal growth factor receptor 2 (HER2) (p = 0.0225) **(D)** and Proliferating cell nuclear antigen (PCNA) **(E)**. Histograms represent the means ± SEM (n = 10 pups, 1 per litter) for each band normalized to the total protein level. p-values were calculated with a Kruskal-Wallis statistical test or ANOVA. Oil-treated animals are showed in **Supplementary Figures**.

**Figure 3:**
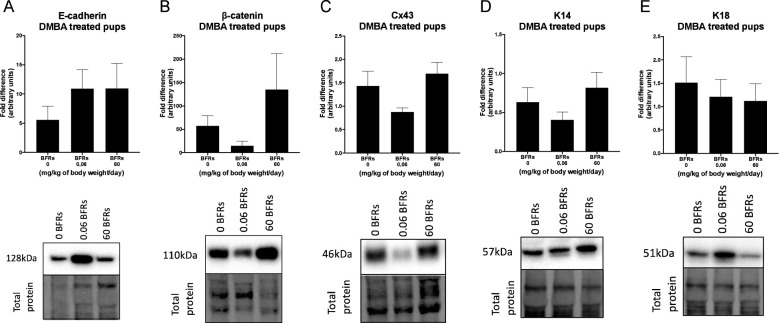
Effects of exposure *in utero* and through lactation to a brominated flame retardant (BFR) mixture and DMBA-treated on junctional and keratin protein levels of female pups. Semi-quantitative Western Blot analysis of total proteins extracted from the mammary tumors after indirect exposure to 0, 0.06 or 60 mg/kg of body weight/day and gavage by DMBA at PND46. Graphs show average expression of E-cadherin **(A)**, β-catenin **(B)**, connexin-43 (Cx43) **(C)**, keratin-14 (K14) **(D)** and keratin-18 (K18). **(E)**. Histograms represent the means ± SEM (n = 6-10 pups, 1 per litter) for each band normalized to the total protein level. p-values were calculated with a Kruskal-Wallis statistical test or ANOVA. Oil-treated animals are showed in **Supplementary Figures**.

We then characterize DMBA-induced tumors in all treatment groups, as well as the effects of the BFRs treatments, on protein expression. Although some variations could be observed, only the expression of ERα was affected by the high dose of BFRs in the mammary glands of rats not exposed to DMBA (**Supplementary Figures 3**, **4**). In addition, PCNA and HER2 were not visible by western blot in the oil-treated group. Globally, we observed changes in protein expression when comparing tumors from DMBA-exposed rats to mammary gland extracts from rats not exposed to DMBA. A decrease in the ERβ was observed, while levels of ERα, the PR A/B, HER2, PCNA, E-cadherin and β-catenin all increased in tumors from DMBA-treated rats compared to mammary gland from controls most of these changes being significant (**Figures 2**, **3**; **Supplementary Figures 3**, **4**).

**Figure 4:**
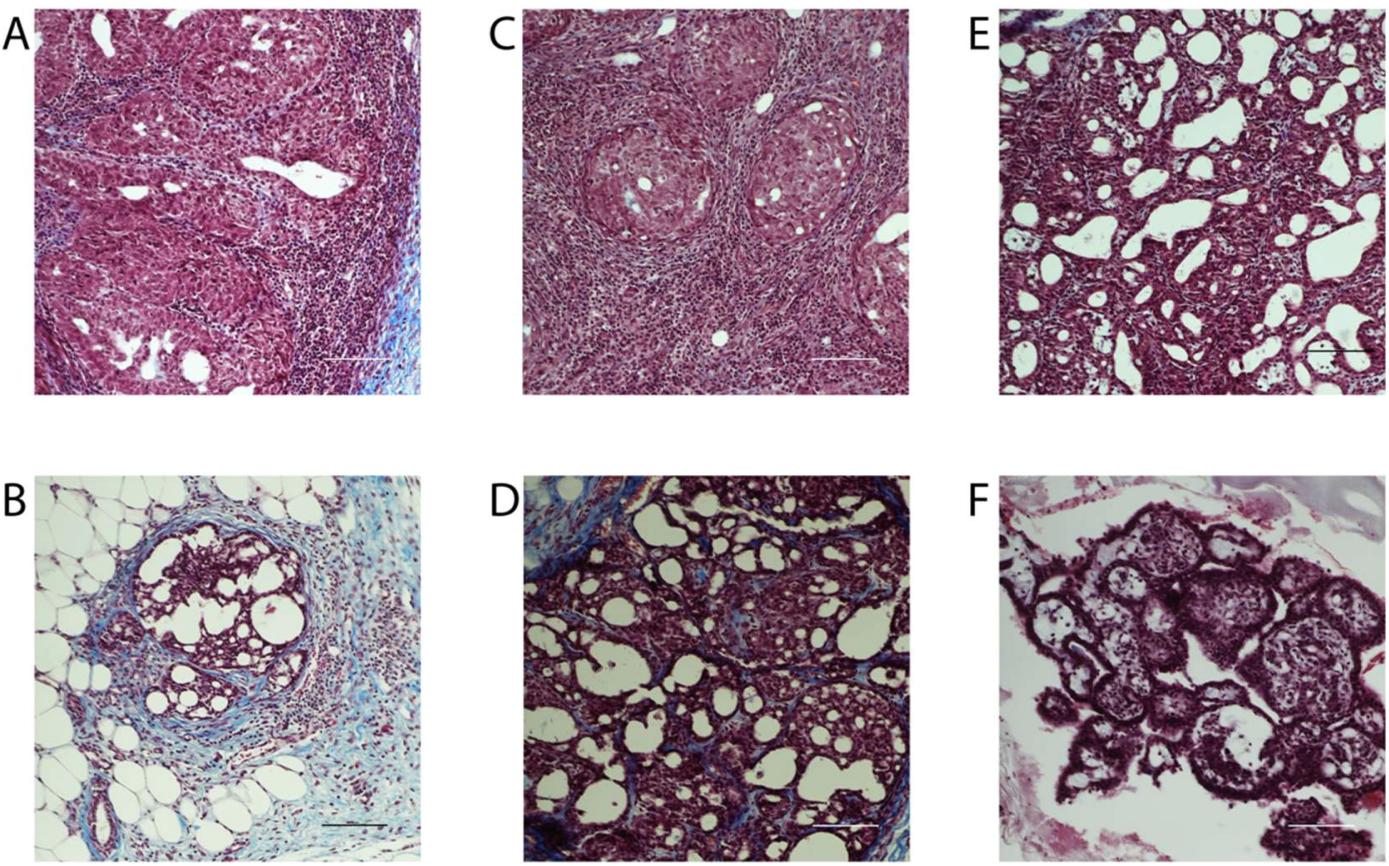
Histology of mammary tumors using Masson's trichome staining. Exposure to BFRs did not affect the histological class of breast cancer. Cribriform carcinoma was the most represented breast cancer in the animals of the study independently to the exposure to BFRs. Images of representative tumors from DMBA-treated female pups upon gestational-lactational exposure to a mixture of BFRs at 0 mg/kg/day **(A, B)**, 0.06 mg/kg/day **(C, D)** and 60 mg/kg/day **(E, F)**. Tumors, excised at necropsy, were formalin fixed and paraffin embedded, and 5 µm sections were stained with Masson's trichrome. Scale = 100 μm.

Breast cancer can be classified histologically but can also be divided into molecular subtypes characterized by the expression of markers, which are related to different prognosis and responses to therapies (**Supplementary Figure 6**). We thus aimed to characterize tumor subtypes that were induced by DMBA and exposure to BFRs. First, tissue sections were analyzed blindly by a pathologist and no differences were observed when comparing the tumors as most were cribriform *in situ* carcinomas (**Figure 4**). Then, the expression of markers of mammary cancer subtypes was assessed using tissue homogenates. When comparing the expression of the protein markers between rats treated with DMBA, no difference was observed for ERs, PRs and PCNA. A significant decrease of HER2, a marker associated with HER2-enriched tumors, was observed in the group treated with the low dose of BFRs in comparison to the control group, while the decrease was not significant in high-dose BFRs treatment (**Figure 2D**).

### 3.4 BFRs treatments might affect the distribution of the subtypes of mammary cancer tumors

**Figure 5:**
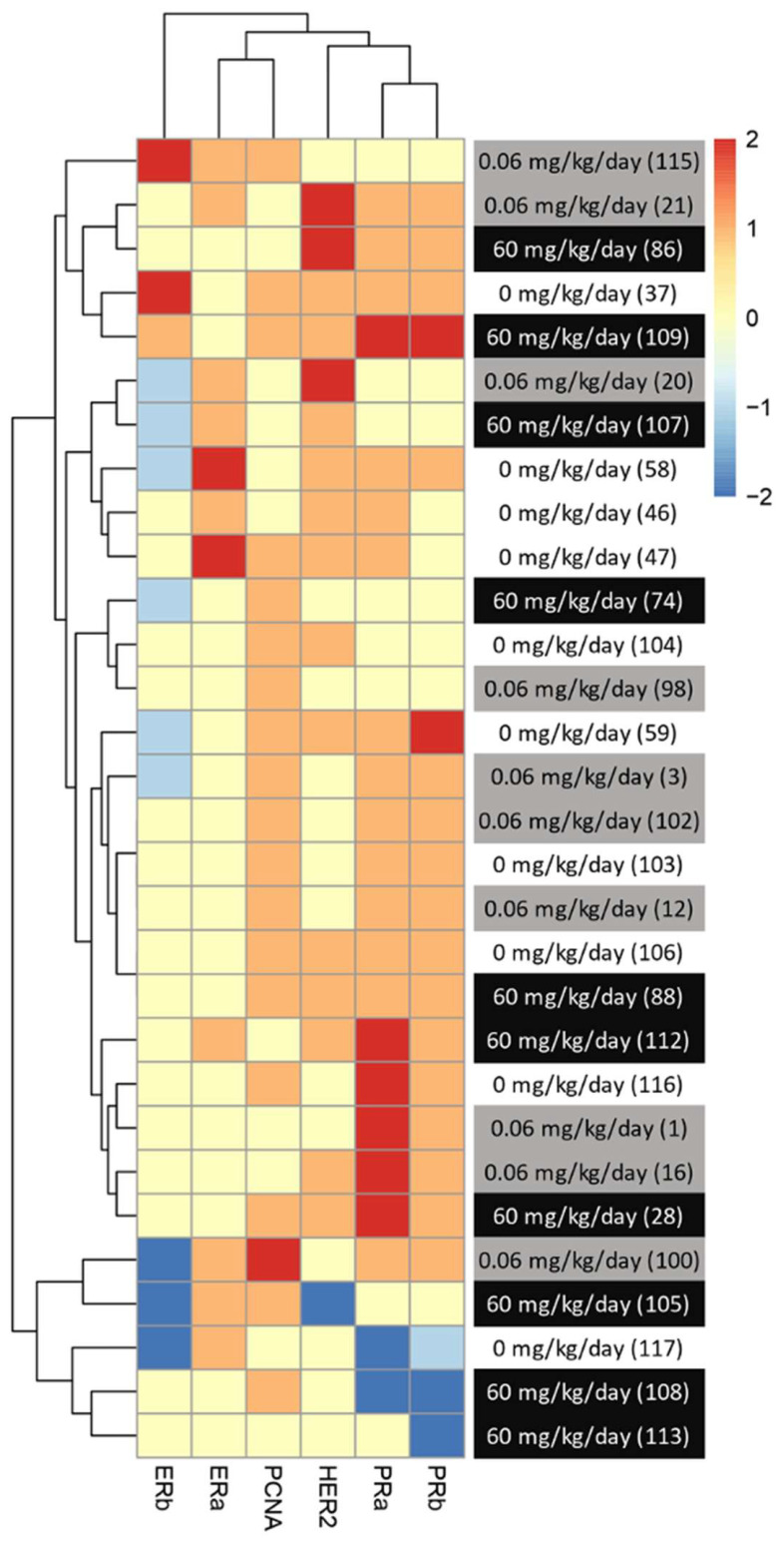
Visualization of clustered protein expression profile per pup. Clustered heatmap of markers for breast cancer subtype classification among the animals from different treatments (0, 0.06 and 60 mg/kg of body weight/day). Each line represents relative levels of the markers for each animal (identified by the numbers in brackets). The interval of protein expression of animals from the group exposed to 0 mg/kg of body weight/day and given oil by gavage at PND46 was considered as a for this heatmap. The graph was coded with the pheatmap Rstudio function (n = 10 pups, 1 per litter).

Although the average levels of expression were not significantly changed between treatment groups for most of the makers observed, trends could be observed when all the changed in hormonal receptors were analyzed together. In addition, variability could be observed between animals from the same group. Since DMBA-induced mammary cancer is known for its heterogenic type of tumors, we then analyze potential changes in the subtype of mammary cancer based on individual protein profiles, rather than an analysis per treatment group. To do so, we created a heatmap with an increase and decrease in protein expression in comparison to the control groups for each animal within the groups. The level of expression of the group exposed to neither BFRs nor DMBA was considered 0 for this heatmap (**Figure 5**).

**Figure 6:**
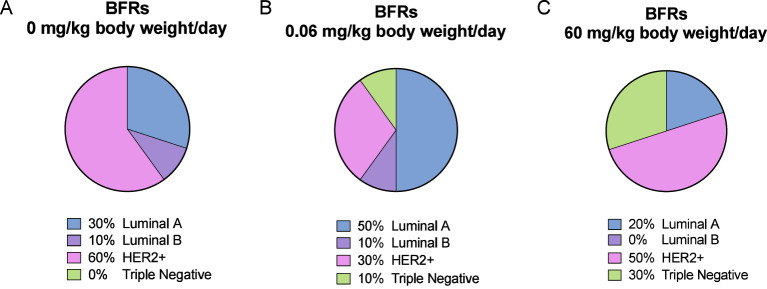
Visualization of breast cancer subtypes based on their protein expression. The classification was performed based on **Figure 5** profiles and **Supplementary Figure S6** description. DMBA-induced tumors from animals exposed to control diet were composed of 30% luminal A, 10% luminal B, 60% HER2-enriched and 0% Triple-Negative (TN) subtypes **(A)**. For the low dose of BFRs (0.06 mg/kg of body weight/day), an increase in the presence of TN (10%) and of luminal A, and a decrease of HER2-enriched subtypes were observed, while luminal B percentage remained unchanged **(B)**. In animals exposed to the high dose of the BFRs mixture (60mg/kg body weight/day), there was an increase of TN, and a slight decrease of HER2-enriched and luminal A tumors percentages compared to control; no luminal B subtype tumors were identified **(C)**.

We noticed a change in the distribution of the subtypes of mammary cancer for the BFRs exposed group in comparison to the control group. In the control group, the induction of mammary cancer by DMBA resulted in about 30% luminal A, 10% luminal B, 60% HER2-enriched and no cases of triple-negative tumors (TNBC), classified solely on the base of the levels of expression of ER, PR, HER2 and PCNA (**Figure 6**). However, in the group exposed to 0.06 mg/kg of body weight/day, the distribution presented 50% luminal A, 10% luminal B, 30% HER2-enriched and 10% TNBC. Finally, when exposed to the highest dose of BFRs, 60mg/kg body weight/day, Luminal A corresponded to 20%, HER2-enriched 50% and TNBC increased to 30% while no luminal B tumors were present. These results suggest that exposure to BFRs might result in a shift in the distribution of mammary cancer subtypes, showing, in this case, an increased proportion of TNBC tumors, and a decrease mainly in the HER2-enriched tumors. It has to be noted that statistical analysis could not be performed due to the size of the sample used.

### 3.5 BFRs exposure does not seem to affect the presence of metastasis in the lungs but alters their histology

**Figure 7:**
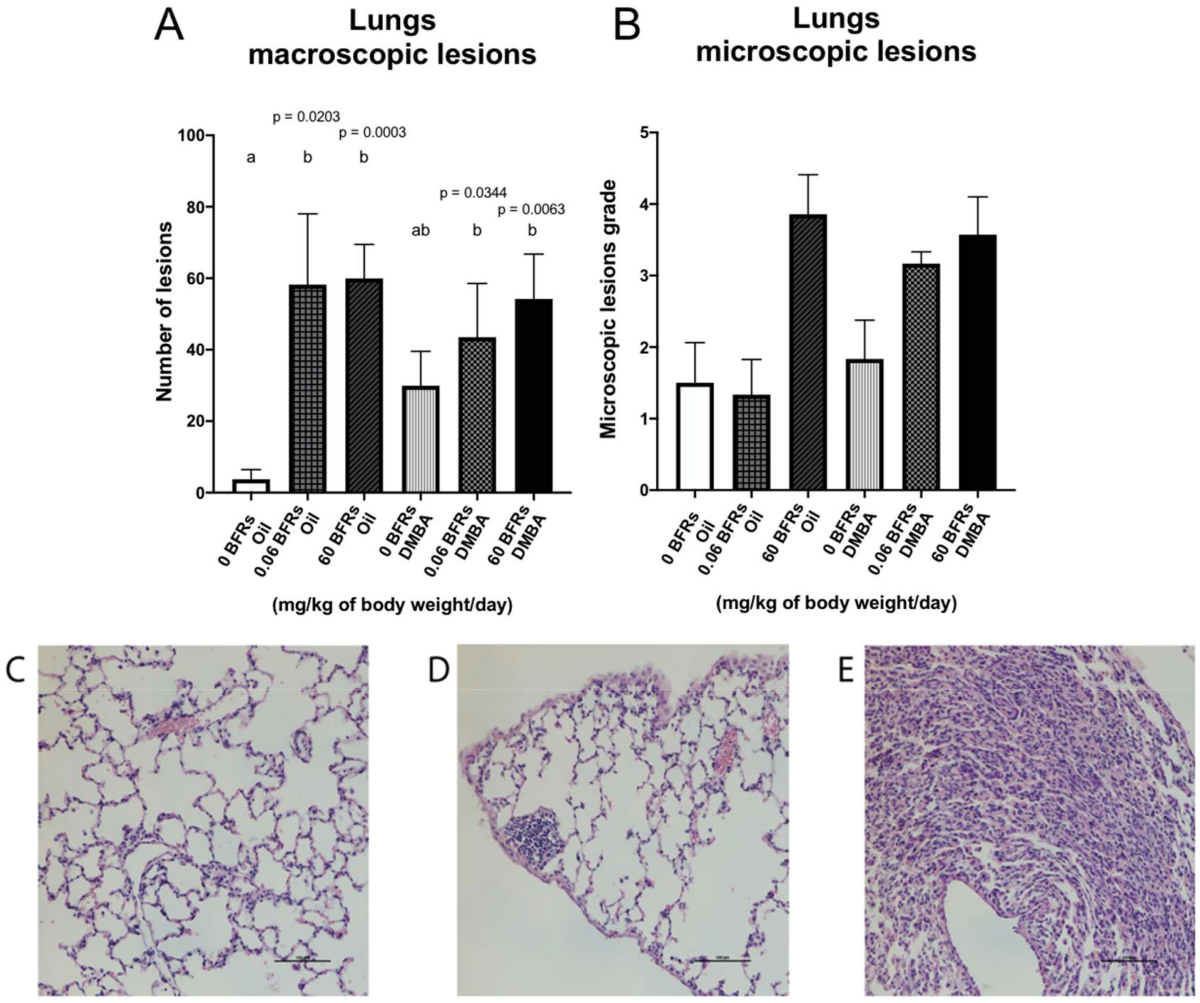
Representation of lung lesions after BFRs exposure *in utero* and through lactation. The animals exposed to BFRs showed macroscopic and microscopic lesions, resulting in a significant difference between BFRs-exposed and control groups for the macroscopic lesions **(A, B)**. Quantitative analysis of microscopic lesions was done by giving a grade to each slide [0 normal tissue **(C)**, 1 abnormal cell accumulation **(D)** and 3 = lesions **(E)**]. Histograms represent the means ± SEM (n ≥ 6 pups, 1 per litter). p-values were calculated with a Kruskal-Wallis statistical test or ANOVA. Variables are statistically indistinguishable if they share at least one letter. Scale = 100 µm.

Lungs are one of the main sites for breast cancer metastasis (63); in clinic, 60 -70% of patients who eventually die from breast cancer have metastasis in their lungs (64). Therefore, at the time of the necropsy, we harvested the lungs and visible lesions were counted. We noticed a significant increase of macroscopic lesions in the lungs of animals exposed to BFRs in both DMBA and oil treated groups suggesting that the lesions were the result of BFRs exposure rather than DMBA treatment (**Figure 7A**). We then stained lung sections using hematoxylin and eosin to assess tissue structure. Lesions tended to consist of collections of small cells with basophilic nuclei and without obvious thickening of the alveolar epithelium. The microscopic lesions in lung samples were assessed for severity using a semiquantitative scale in which a value was given after analysis by a blinded observer (0 = normal tissue, 1 = abnormal cell accumulation, 3 = lesions) (**Figures 7C–E**). Although no significant differences were found among the groups, the same trend was observed for microscopic lesions as for macroscopic lesions for the BFRs exposed groups (**Figure 7B**). To determine whether some mammary cancer cells could be present within the lungs, lung sections were then stained with mammaglobin, a marker known to identify breast cancer metastasis (65); sections stained were given a score based on the presence of mammaglobin (absent = 0, present = 1). There was no significant difference in the presence of mammaglobin in the lung tissue, positive cases were found in all the DMBA groups, showing that BFRs do not increase the risk of developing lung metastasis (**Supplementary Figure 5**). Additionally, the images were blindly analyzed by a pathologist, and the non-cancerous nature of these lesions were confirmed; lesions were remiscent of inflammation within the lungs.

## 4 Discussion

Our previous studies on the effects of gestational-lactational exposure to BFRs resulted in a dysregulation of proliferation-apoptosis balance, cell-cell communication and a precocious mammary gland development, all associated with increased risk for mammary cancer (50, 51). In the present study, we thus examined the effects of this same gestational-lactational exposure to BFRs on the progression of mammary gland tumors induced by DMBA at PND46. DMBA is a polycyclic aromatic hydrocarbon (PAH) well known for its capacity to induce mammary cancer in female Sprague-Dawley rats (66). It has been shown that a single dose of DMBA at a peri-pubertal stage is 100% efficient to induce mammary cancer in rats (67), likely due to the presence of numerous TEBs in the proliferating epithelium at this age (68). Furthermore, rodent DMBA-induced tumors are heterogenic in their molecular subtypes (69) and they closely mimic the multistep process of the development of human breast cancer (initiation, promotion and progression; reviewed by (70). Even though mice mutant models are known to be used for the research of breast cancer, we decided to use Sprague Dawley rats and the same protocol of exposure to reproduce our previous studies but this time in a context of mammary cancer.

In this study, one dose of DMBA (20 mg) at PND46 showed 100% efficiency in the control group, and 50% of the rats had at least one palpable tumor 50 days after exposure. These tumors were heterogenic, and they represented 3 out of 4 subtypes of mammary cancer based on their protein expression profile, as no TNBC tumors were observed in DMBA-treated females not exposed to the BFR mixture. Surprisingly, exposure to BFRs during gestation and through lactation caused a delay in the onset and slower growth of DMBA-induced tumors in rats. Three non-exclusive hypotheses could explain these results. First, it is possible that exposure to the mixture of BFRs induced changes in the programming of the cells, modifying the response of the cells to DMBA favoring the TNBC and HER2-enriched subtype, or inhibiting the luminal subtype. Second, it has been shown that exposure to the BFR mixture during pregnancy and lactation increases the activity of a variety of CYP enzymes in the livers (28, 45) and this elevation was still apparent at PND 46 (Tung et al., 2016). It is possible that the liver capacity to metabolize DMBA was altered in BFR-exposed animals resulting in an altered distribution of reactive metabolites compared to control animals, thus modifying the response of the mammary gland at puberty. Third, it has been demonstrated that the sensitivity of the rat mammary gland to DMBA is optimal at PND46 when several TEBs are present (68). Given that our previous work suggests that BFR-exposure may accelerate some aspects of mammary gland development, it is possible that the shift in phenotype may alter the sensitivity/response to DMBA-induced tumorigenesis.

### 4.1 BFRs-induced changes in signaling could represent a shift in the distribution of change tumor subtypes

A potential explanation for the delayed tumor onset and slower growth of the tumors was a change in tumor subtypes. Histologically, breast tumors can be classified by stage and grade, as well as histologic features (as reviewed by (71). When tumors were evaluated blindly by a pathologist, no differences were noted in the types of tumors between groups, most of them being cribriform carcinomas, often associated with Ductal carcinoma *in situ* (DCIS) (71) (**Figure 4**). However, breast cancer is a heterogeneous disease that encompasses many subtypes with various prognostic, responses to therapies, aggressiveness and molecular signatures (72–74). Luminal A tumors express ER and/or PR while being HER2 negative (**Supplementary Figure 6**). Luminal B tumors have a worse clinical prognosis compared to luminal A, also express ER and can be PR negative. Luminal B tumors are also generally HER2 negative (reviewed by (72). HER2-enriched are ER and PR negative but overexpress HER2. In this group, two subcategories can be distinguished: luminal HER2 (ER+ and/or PR+ and HER2+) and HER2-enriched (ER-, PR- and HER2+) (75). These subgroups have a worse prognosis than the luminal classification. Finally, TNBC is negative for ER, PR and HER2 (76). Being able to identify the type of cancer allows for a better prognosis and treatment. Therefore, luminal A, luminal B and HER2-enriched subtypes are treated with targeted treatments, while TNBC tumors use chemotherapy as their treatment option and are generally associated with a poorer prognosis (77).

We thus speculate that BFRs, being recognized as EDCs, could dysregulate hormonal pathways and favor the development of tumors from different subtypes. The changes in mammary cancer subtype after exposure to BFRs could also be driven and/or linked to the downregulation in adherens, junctional proteins and apoptosis markers noticed at PND46 (50), which are markers of more aggressive types of breast cancer (41). We performed a rough and simple classification of individual tumors based on the levels of ER, PR, HER2 and PCNA levels, to divide them as luminal A (ER+, low PR+, HER2-), Luminal B (ER+, high PR+, HER2-), HER2-enriched (ER-, PR-, HER2+) or TNBC (ER-, PR-, HER2-). Although a switch from more aggressive HER2-enriched tumors, which represented the majority of tumors in the control group, to more luminal-type tumors in the group treated with the lower dose of BFRs could be observed, we also observed a higher number of TNBC, which are considered the most aggressive tumors. This number was even more important in the group treated with the highest dose of BFRs. Consequently, the decrease in the number of HER2-enriched tumors (from 60% to 30 - 50%) could explain the change in the average tumor onset time or speed of tumor growth. Nevertheless, we would require further analysis to confirm this affirmation, since progression and invasiveness of mammary cancer are not necessarily homologous between Sprague-Dawley rats and humans as reviewed by (78).

### 4.2 Exposure to a mixture of BFRs can affect the metabolism of DMBA at puberty, thus delaying the development of mammary cancer

DMBA is a procarcinogen that requires metabolic activation and results in short-lived unstable carcinogen metabolites (79). The first process includes the activation of the aryl hydrocarbon receptor (AhR) which induces the transcription of genes, including the cytochrome P450 enzymes (CYP1B1 and CYP1A1) (80, 81). These enzymes act on DMBA to form highly reactive metabolites which form DNA-adducts, resulting in mutations and tumorigenesis (80). In addition to DNA-adducts, DMBA-induced changes in intracellular signaling and gene expression dysregulation are believed to contribute to tumor promotion and progression (70, 81, 82).

Even though the liver and lung are considered the major sites for polyaromatic hydrocarbon metabolism (including DMBA), the mammary gland stroma can also accumulate hydrophobic compounds such as the DMBA and can respond to AhR ligands to express CYP1B1 (80). As such, DMBA metabolism may occur within the mammary gland leading to procarcinogen generation in close proximity to the mutagenic target cells (83). After activation, the metabolites interact with the proliferating cells in the TEBs to form DNA-adducts which will later turn into malignant cells (84, 85). DMBA excretion has been shown to be completed after 24 h (86).

A suspected competition between the metabolism of other chemical components and DMBA has been proposed previously (87), causing a reduction in the bioactivation of DMBA to its active carcinogenic metabolite and therefore, a decrease in tumor incidence (88, 89). The phenomena were observed only when exposure to other molecules was done before gavage with DMBA (88). It has been previously shown that other EDCs such as 2,3,7,8-tetrachlorodibenzodioxin (TCDD) can increase the time required for the development of DMBA-initiated mammary tumors in Sprague-Dawley rats probably due to the induction of CYP enzymes prior to DMBA exposure (90). Additionally, it has been shown that BFRs mixtures can activate the AhR as observed with TCDD exposure (91).

Interestingly, activities of 7-ethoxy-resorufin O-deethylase (EROD) and 7-methoxy-resorufin O-deethylase (MROD) – markers of CYP1A1/1A2 and CYP1A1 activity, respectively, were elevated in livers of PND46 female pups exposed to the high dose, but not at the low dose, after a similar exposure *in utero* and lactation to the mixture of BFRs. Similarly, liver hypertrophy was observed in these same high-dose animals at PND21 but not in low-dose treated females at PND21 or animals exposed to either dose at PND46 (45). Furthermore, elevated levels of both HBCDD and PBDEs were still detectable in the livers of pups at PND21 and PND46 (45), meaning that BFRs were likely still present in our study when DMBA was given by gavage. BFR exposure *in utero* and during lactation might have caused either or both an increase in the metabolism and excretion of DMBA, limiting the amount of parent and metabolites reaching the mammary gland tissue. As CYP1 activity was most clearly induced in animals exposed to the high dose in our previous study (45), one might expect altered tumorigenesis due to pre-induction of CYP1 to be evident only in the high-dose animals. The delay in tumor initiation and growth seen in the low-dose animals, although of lesser extent than in the high dose, suggests that additional mechanism(s) may contribute to this delay.

### 4.3 The BFRs-associated precocious mammary gland development may alter the effects of DMBA in mammary cancer induction

The effectiveness of DMBA to induce mammary cancer under normal conditions has been reported to be tightly related to the transition period of TEB evolution (68). It has been shown that there is a sharp decrease in tumor yield when DMBA is given after PND55 because the proliferative activity of the mammary epithelial cells is decreased. Since a malignant transformation is required for carcinogenesis, as the rate of cell division decreases, the risk of malignant transformation follows as well (92). In our study, DMBA was administered orally at PND46 since BFR-induced dysregulation of mammary gland signaling was previously observed at that age (50, 51). Additionally, the peripubertal period is known to be a window of sensibility for chemically induced mammary tumorigenesis (41, 67, 93).

However, it has been shown previously that the window of susceptibility for DMBA might be shifted after exposure to EDCs *in utero* (94). In that study, cancer was induced by a single dose of DMBA after an *in utero* exposure to bisphenol A (BPA), either at PND50 or PND100. The authors showed a higher tumor incidence and decreased tumor latency in rats exposed to BPA compared to the control group when DMBA was administrated at PND100, but not when administrated at PND50 (94). Interestingly, in a previous study, the authors did not observe any difference in the number of TEBs at PND50 in rats treated with BPA using the same protocols but found a higher number of terminal ducts (TDs) at PND100 (95). These results suggest that a change in TEBs or TDs alone could influence the sensitivity of the mammary gland to the inductions of mammary cancer through DMBA. Both structures are considered the most susceptible because of their high mitotic index and undifferentiated cells (96).

In our case, we noticed that after exposure to the low dose of BFRs *in utero* and through lactation, there is a precocious mammary gland development at PND21 as suggested by an increased epithelial surface area, a tendency to increase the ductal area and thickness, and of lumen area, and a significant increase of the Ki67 cell proliferation index and of the early apoptotic marker cleaved caspase-9 (50). These results suggest that, after exposure to a relevant mixture of BFRs *in utero* and through lactation, the mammary gland’s susceptibility window to DMBA may be shifted and possibly happen earlier, at least in the low-dose group. It is thus possible that the increased latency for tumor onset and/or decreased growth of tumors is linked with the timing of tumor induction rather than a change in susceptibility induced by BFRs for the low-dose group. Notably, no difference in any of these markers was observed at the high dose in this study. This result further suggests that delayed tumorigenesis in low dose BFR exposed females may be due to distinct mechanism(s) than that observed at the high dose.

### 4.4 Evidence of the effects of BFRs on mammary cancer

Interest in PBDEs as potential human carcinogens has raised concerns based on their structural similarities and toxicological properties to polychlorinated biphenyls (PCBs), which are now known as human chemical carcinogens (97). However, only a few studies have evaluated the carcinogenicity of BFRs. Deca-BDE caused a significant increase in hepatocellular carcinomas in mice and dose-related increases in liver and pancreatic adenomas in rats (98). Some epidemiology studies have reported increased risks for testicular cancer (99), as well as childhood acute lymphoblastic leukemia (100) due to BFR exposure. A Chinese case-control study observed that significantly elevated adipose tissue levels of multiple PBDE congeners were associated with breast cancer diagnoses (43). Further, PBDE exposure in ovariectomized estrogen-treated mice led to mammary gland remodeling and enhanced response to estrogen signaling (31).

Further interest in the effect of BFRs in the development of breast cancer is being driven by previous evidence showing the endocrine-disrupting effects of PBDEs. These effects include the ability of BFR to alter the *in vivo* circulating sex hormone concentrations (98), to interact with estrogen signaling pathways and to enhance estrogenic-related cellular proliferation in breast cancerous cell line MDA-MB-231 (101) and MCF-7 (102). It has been demonstrated that PBDEs can behave as an agonist for ERα and ERβ in the T47D breast cancerous cell line (35). One PBDE congener (BDE-209) was evaluated by the United States Environmental Protection Agency (US EPA) in 2008 and they concluded that there was “suggestive evidence of human carcinogenic potential” (103).

### 4.5 BFRs exposure *in utero* and through lactation creates lung lesions independent of the development of mammary cancer

Macroscopic and microscopic lesions were identified in both healthy and tumor-bearing animals. These lesions do not appear to be related to metastatic mammary gland tumors but appear to be aggregations of non-epithelial cells. In collaboration with Dr Gaboury, we hypothesize that these structures are collections of various immune cell types in the lung related directly to the exposure to BFRs and not related to the metastasis from mammary cancer. It has been previously reported that exposure to some BFRs, such as PBDEs, can cause oxidative stress, inflammatory response and changes in junctional proteins in lung epithelial cells *in vitro* (104). Additionally, exposure to HBCDDs can disrupt the expression of proinflammatory proteins in bronchial epithelial cells (105). Pups were mainly exposed *in utero* and through lactation to the mixture of BFRs, but they could have been exposed to BFRs by inhalation from the food dust from birth to weaning at PND21. Inflammatory effects of BFRs in the lungs would, therefore, last until adulthood without direct exposure to BFRs. Further studies are needed to characterize the nature of these observed lung lesions.

## 5 Conclusion

Overall, our results show that exposure to a representative mixture of BFRs *in utero* and through lactation results in a reduced pace of DMBA-induced tumorigenesis and a possible change in the profile of the molecular subtype of mammary tumors developed. The profile presented in the low and high doses of BFRs appears to correspond to a more aggressive type of mammary cancer (TNBC and HER2-enriched). Nevertheless, tumor onset and latency were significantly delayed compared with the control group. These phenomena might be explained by molecular dysregulations during early mammary gland development and a shift in the susceptibility window after exposure to BFRs. Further research is required to confirm the shift of susceptibility window to DMBA and the mechanisms involved in the variation of mammary cancer subtypes. Additionally, mechanisms bringing these results might be different at low and high dose, as results in tumor delay and subtypes of mammary cancer does not follow a monotonic dose-response situation. Further analysis into the effect of gestational-lactational exposure to BFRs in the development of mammary cancer could be confirmed using a different model, as DMBA-induced cancer showed to present a complexity by itself and its interaction with EDCs.

## Data Availability

The original contributions presented in the study are included in the article/**Supplementary Material**. Further inquiries can be directed to the corresponding author.

## References

[1] Janssen S. Brominated flame retardants: rising levels of concern. Arlington, VA: Health Care Without Harm (2005).

[2] Birnbaum LS Staskal DF. Brominated flame retardants: cause for concern? Environ Health Perspect. (2004) 112:9–17. doi: 10.1289/ehp.6559 14698924 PMC1241790

[3] Alaee M Arias P Sjodin A Bergman A. An overview of commercially used brominated flame retardants, their applications, their use patterns in different countries/regions and possible modes of release. Environ Int. (2003) 29:683–9. doi: 10.1016/S0160-4120(03)00121-1 12850087

[4] Alaee M Wenning RJ. The significance of brominated flame retardants in the environment: current understanding, issues and challenges. Chemosphere. (2002) 46:579–82. doi: 10.1016/S0045-6535(01)00224-7 11999783

[5] Remberger M Sternbeck J Palm A Kaj L Stromberg K Brorstrom-Lunden E. The environmental occurrence of hexabromocyclododecane in Sweden. Chemosphere. (2004) 54:9–21. doi: 10.1016/S0045-6535(03)00758-6 14559253

[6] Hale RC Alaee M Manchester-Neesvig JB Stapleton HM Ikonomou MG. Polybrominated diphenyl ether flame retardants in the North American environment. Environ Int. (2003) 29:771–9. doi: 10.1016/S0160-4120(03)00113-2 12850095

[7] Stapleton HM Dodder NG Offenberg JH Schantz MM Wise SA. Polybrominated diphenyl ethers in house dust and clothes dryer lint. Environ Sci Technol. (2005) 39:925–31. doi: 10.1021/es0486824 15773463

[8] La AGMJ Hale RC Harvey E. Detailed polybrominated diphenyl ether (PBDE) congener composition of the widely used penta-, octa-, and deca-PBDE technical flame-retardant mixtures. Environ Sci Technol. (2006) 40:6247–54. doi: 10.1021/es060630m 17120549

[9] Allen JG McClean MD Stapleton HM Webster TF. Critical factors in assessing exposure to PBDEs via house dust. Environ Int. (2008) 34:1085–91. doi: 10.1016/j.envint.2008.03.006 18456330

[10] EU. REGULATION (EU) 2019/1021 OF THE EUROPEAN PARLIAMENT AND OF THE COUNCIL official journal of the European union: official journal of the European union(2019). Available online at: https://eur-lex.europa.eu/legal-content/en/TXT/?uri=CELEX%3A32019R1021.

[11] UNEP. Notifications of articles in use 2021(2023). Available online at: https://chm.pops.int/Implementation/Exemptions/Articlesinuse/tabid/452/Default.aspx.

[12] Sharkey M Harrad S Abou-Elwafa Abdallah M Drage DS Berresheim H. Phasing-out of legacy brominated flame retardants: The UNEP Stockholm Convention and other legislative action worldwide. Environ Int. (2020) 144:106041. doi: 10.1016/j.envint.2020.106041 32822924

[13] Schecter A Pavuk M Papke O Ryan JJ Birnbaum L Rosen R. Polybrominated diphenyl ethers (PBDEs) in U.S. mothers' milk. Environ Health Perspect. (2003) 111:1723–9. doi: 10.1289/ehp.6466 PMC1241714 14594622

[14] Butt CM Diamond ML Truong J Ikonomou MG ter Schure AF. Spatial distribution of polybrominated diphenyl ethers in southern Ontario as measured in indoor and outdoor window organic films. Environ Sci Technol. (2004) 38:724–31. doi: 10.1021/es034670r 14968856

[15] Wilford BH Harner T Zhu J Shoeib M Jones KC. Passive sampling survey of polybrominated diphenyl ether flame retardants in indoor and outdoor air in Ottawa, Canada: implications for sources and exposure. Environ Sci Technol. (2004) 38:5312–8. doi: 10.1021/es049260x 15543731

[16] Rudel RA Camann DE Spengler JD Korn LR Brody JG. Phthalates, alkylphenols, pesticides, polybrominated diphenyl ethers, and other endocrine-disrupting compounds in indoor air and dust. Environ Sci Technol. (2003) 37:4543–53. doi: 10.1021/es0264596 14594359

[17] Fraser AJ Webster TF McClean MD. Diet contributes significantly to the body burden of PBDEs in the general U.S. population. Environ Health Perspect. (2009) 117:1520–5. doi: 10.1289/ehp.0900817 PMC2790504 20019900

[18] Bocio A Llobet JM Domingo JL Corbella J Teixido A Casas C. Polybrominated diphenyl ethers (PBDEs) in foodstuffs: human exposure through the diet. J Agric Food Chem. (2003) 51:3191–5. doi: 10.1021/jf0340916 12720414

[19] Ionas AC Ulevicus J Gomez AB Brandsma SH Leonards PE van de Bor M. Children's exposure to polybrominated diphenyl ethers (PBDEs) through mouthing toys. Environ Int. (2016) 87:101–7. doi: 10.1016/j.envint.2015.11.018 26655676

[20] Linares V Belles M Domingo JL. Human exposure to PBDE and critical evaluation of health hazards. Arch Toxicol. (2015) 89:335–56. doi: 10.1007/s00204-015-1457-1 25637414

[21] Vuong AM Braun JM Webster GM Thomas Zoeller R Hoofnagle AN Sjodin A. Polybrominated diphenyl ether (PBDE) exposures and thyroid hormones in children at age 3 years. Environ Int. (2018) 117:339–47. doi: 10.1016/j.envint.2018.05.019 PMC5997562 29787984

[22] Wang X Hales BF Robaire B. Effects of flame retardants on ovarian function. Reprod Toxicol. (2021) 102:10–23. doi: 10.1016/j.reprotox.2021.03.006 33819575

[23] Ermler S Kortenkamp A. Declining semen quality and polybrominated diphenyl ethers (PBDEs): Review of the literature to support the derivation of a reference dose for a mixture risk assessment. Int J Hyg Environ Health. (2022) 242:113953. doi: 10.1016/j.ijheh.2022.113953 35334436

[24] Gibson EA Siegel EL Eniola F Herbstman JB Factor-Litvak P. Effects of polybrominated diphenyl ethers on child cognitive, behavioral, and motor development. Int J Environ Res Public Health. (2018) 15. doi: 10.3390/ijerph15081636 PMC6121413 30072620

[25] Vuong AM Yolton K Dietrich KN Braun JM Lanphear BP Chen A. Exposure to polybrominated diphenyl ethers (PBDEs) and child behavior: Current findings and future directions. Horm Behav. (2018) 101:94–104. doi: 10.1016/j.yhbeh.2017.11.008 29137973

[26] Zhang T Zhou X Xu A Tian Y Wang Y Zhang Y. Toxicity of polybrominated diphenyl ethers (PBDEs) on rodent male reproductive system: A systematic review and meta-analysis of randomized control studies. Sci Total Environ. (2020) 720:137419. doi: 10.1016/j.scitotenv.2020.137419 32325560

[27] Gouesse RJ Plante I. Environmental exposure to brominated flame retardants: unraveling endocrine and mammary gland effects that may increase disease risk. Toxicol Sci. (2022) 186:190–207. doi: 10.1093/toxsci/kfac006 35104882

[28] Ernest SR Wade MG Lalancette C Ma YQ Berger RG Robaire B. Effects of chronic exposure to an environmentally relevant mixture of brominated flame retardants on the reproductive and thyroid system in adult male rats. Toxicol Sci. (2012) 127:496–507. doi: 10.1093/toxsci/kfs098 22387749 PMC3355309

[29] Kodavanti PR Coburn CG Moser VC MacPhail RC Fenton SE Stoker TE. Developmental exposure to a commercial PBDE mixture, DE-71: neurobehavioral, hormonal, and reproductive effects. Toxicol Sci. (2010) 116:297–312. doi: 10.1093/toxsci/kfq105 20375078

[30] Cao LY Ren XM Yang Y Wan B Guo LH Chen D. Hydroxylated polybrominated diphenyl ethers exert estrogenic effects via non-genomic G protein-coupled estrogen receptor mediated pathways. Environ Health Perspect. (2018) 126:057005. doi: 10.1289/EHP2387 29790728 PMC6071991

[31] Kanaya N Chang G Wu X Saeki K Bernal L Shim HJ. Single-cell RNA-sequencing analysis of estrogen- and endocrine-disrupting chemical-induced reorganization of mouse mammary gland. Commun Biol. (2019) 2:406. doi: 10.1038/s42003-019-0618-9 31701034 PMC6831695

[32] Stoker TE Cooper RL Lambright CS Wilson VS Furr J Gray LE. *In vivo* and *in vitro* anti-androgenic effects of DE-71, a commercial polybrominated diphenyl ether (PBDE) mixture. Toxicol Appl Pharmacol. (2005) 207:78–88. doi: 10.1016/j.taap.2005.05.010 16005038

[33] Yamada-Okabe T Sakai H Kashima Y Yamada-Okabe H. Modulation at a cellular level of the thyroid hormone receptor-mediated gene expression by 1,2,5,6,9,10-hexabromocyclododecane (HBCD), 4,4'-diiodobiphenyl (DIB), and nitrofen (NIP). Toxicol Lett. (2005) 155:127–33. doi: 10.1016/j.toxlet.2004.09.005 15585367

[34] Legler J Brouwer A. Are brominated flame retardants endocrine disruptors? Environ Int. (2003) 29:879–85. doi: 10.1016/S0160-4120(03)00104-1 12850103

[35] Meerts IA Letcher RJ Hoving S Marsh G Bergman A Lemmen JG. *In vitro* estrogenicity of polybrominated diphenyl ethers, hydroxylated PDBEs, and polybrominated bisphenol A compounds. Environ Health Perspect. (2001) 109:399–407. doi: 10.1289/ehp.01109399 11335189 PMC1240281

[36] Hinck L Silberstein GB. Key stages in mammary gland development: the mammary end bud as a motile organ. Breast Cancer Res. (2005) 7:245–51. doi: 10.1186/bcr1331 PMC1410762 16280048

[37] Paine IS Lewis MT. The terminal end bud: the little engine that could. J Mammary Gland Biol Neoplasia. (2017) 22:93–108. doi: 10.1007/s10911-017-9372-0 28168376 PMC5488158

[38] Sternlicht MD Kouros-Mehr H Lu P Werb Z. Hormonal and local control of mammary branching morphogenesis. Differentiation. (2006) 74:365–81. doi: 10.1111/j.1432-0436.2006.00105.x PMC2580831 16916375

[39] Macias H Hinck L. Mammary gland development. Wiley Interdiscip Rev Dev Biol. (2012) 1:533–57. doi: 10.1002/wdev.v1.4 PMC3404495 22844349

[40] Plante I Winn LM Vaillancourt C Grigorova P Parent L. Killing two birds with one stone: Pregnancy is a sensitive window for endocrine effects on both the mother and the fetus. Environ Res. (2022) 205:112435. doi: 10.1016/j.envres.2021.112435 34843719

[41] Fenton SE Reed C Newbold RR. Perinatal environmental exposures affect mammary development, function, and cancer risk in adulthood. Annu Rev Pharmacol Toxicol. (2012) 52:455–79. doi: 10.1146/annurev-pharmtox-010611-134659 PMC3477544 22017681

[42] Hassiotou F Geddes D. Anatomy of the human mammary gland: Current status of knowledge. Clin Anat. (2013) 26:29–48. doi: 10.1002/ca.22165 22997014

[43] He Y Peng L Zhang W Liu C Yang Q Zheng S. Adipose tissue levels of polybrominated diphenyl ethers and breast cancer risk in Chinese women: A case-control study. Environ Res. (2018) 167:160–8. doi: 10.1016/j.envres.2018.07.009 30014897

[44] Dianati E Wade MG Hales BF Robaire B Plante I. From the cover: exposure to an environmentally relevant mixture of brominated flame retardants decreased p-beta-cateninser675 expression and its interaction with E-cadherin in the mammary glands of lactating rats. Toxicol Sci. (2017) 159:114–23. doi: 10.1093/toxsci/kfx123 PMC5837432 28903489

[45] Tung EW Yan H Lefevre PL Berger RG Rawn DF Gaertner DW. Gestational and early postnatal exposure to an environmentally relevant mixture of brominated flame retardants: general toxicity and skeletal variations. Birth Defects Res B Dev Reprod Toxicol. (2016) 107:157–68. doi: 10.1002/bdrb.2016.107.issue-3 27286044

[46] Lefevre PL Berger RG Ernest SR Gaertner DW Rawn DF Wade MG. Exposure of female rats to an environmentally relevant mixture of brominated flame retardants targets the ovary, affecting folliculogenesis and steroidogenesis. Biol Reprod. (2016) 94:9. doi: 10.1095/biolreprod.115.134452 26607716 PMC4809562

[47] Berger RG Lefevre PL Ernest SR Wade MG Ma YQ Rawn DF. Exposure to an environmentally relevant mixture of brominated flame retardants affects fetal development in Sprague-Dawley rats. Toxicology. (2014) 320:56–66. doi: 10.1016/j.tox.2014.03.005 24670387

[48] Tung EWY Kawata A Rigden M Bowers WJ Caldwell D Holloway AC. Gestational and lactational exposure to an environmentally-relevant mixture of brominated flame retardants: effects on neurodevelopment and metabolism. Birth Defects Res. (2017) 109:497–512. doi: 10.1002/bdr2.v109.7 28398660 PMC5434666

[49] Allais A Albert O Lefevre PLC Wade MG Hales BF Robaire B. *In utero* and lactational exposure to flame retardants disrupts rat ovarian follicular development and advances puberty. Toxicol Sci. (2020) 175:197–209. doi: 10.1093/toxsci/kfaa044 32207525 PMC7253202

[50] Gouesse RJ Lavoie M Dianati E Wade M Hales B Robaire B. Gestational and lactational exposure to an environmentally-relevant mixture of brominated flame retardants down-regulates junctional proteins, thyroid hormone receptor alpha1 expression and the proliferation-apoptosis balance in mammary glands post puberty. Toxicol Sci. (2019) 1:13–31. doi: 10.1093/toxsci/kfz147 PMC6735962 31241157

[51] Gouesse RJ Dianati E McDermott A Wade MG Hales B Robaire B. *In Utero* and lactational exposure to an environmentally relevant mixture of brominated flame retardants induces a premature development of the mammary glands. Toxicol Sci. (2020) 2:206–19. doi: 10.1093/toxsci/kfaa176 33252648

[52] Rudel RA Fenton SE Ackerman JM Euling SY Makris SL. Environmental exposures and mammary gland development: state of the science, public health implications, and research recommendations. Environ Health Perspect. (2011) 119:1053–61. doi: 10.1289/ehp.1002864 PMC3237346 21697028

[53] Carraway KL Ramsauer VP Carraway CA. Glycoprotein contributions to mammary gland and mammary tumor structure and function: roles of adherens junctions, ErbBs and membrane MUCs. J Cell Biochem. (2005) 96:914–26. doi: 10.1002/jcb.20612 16167329

[54] Hatsell S Rowlands T Hiremath M Cowin P. Beta-catenin and Tcfs in mammary development and cancer. J Mammary Gland Biol Neoplasia. (2003) 8:145–58. doi: 10.1023/A:1025944723047 14635791

[55] Dolled-Filhart M McCabe A Giltnane J Cregger M Camp RL Rimm DL. Quantitative *in situ* analysis of beta-catenin expression in breast cancer shows decreased expression is associated with poor outcome. Cancer Res. (2006) 66:5487–94. doi: 10.1158/0008-5472.CAN-06-0100 16707478

[56] Fenton SE. Endocrine-disrupting compounds and mammary gland development: early exposure and later life consequences. Endocrinology. (2006) 147:s18–24. doi: 10.1210/en.2005-1131 16690811

[57] Stapleton HM Allen JG Kelly SM Konstantinov A Klosterhaus S Watkins D. Alternate and new brominated flame retardants detected in U.S. house dust. Environ Sci Technol. (2008) 42:6910–6. doi: 10.1021/es801070p 18853808

[58] Poon S Wade MG Aleksa K Rawn DF Carnevale A Gaertner DW. Hair as a biomarker of systemic exposure to polybrominated diphenyl ethers. Environ Sci Technol. (2014) 48:14650–8. doi: 10.1021/es502789h 25387207

[59] Clemens AM Lenschow C Beed P Li L Sammons R Naumann RK. Estrus-cycle regulation of cortical inhibition. Curr Biol. (2019) 29:605–15.e6. doi: 10.1016/j.cub.2019.01.045 30744972

[60] Plante I. (2021). Dimethylbenz(a)anthracene-induced mammary tumorigenesis in mice. Methods Cell Biol. 163:21–44. doi: 10.1016/bs.mcb.2020.09.003 33785167

[61] Plante I Stewart MK Barr K Allan AL Laird DW. Cx43 suppresses mammary tumor metastasis to the lung in a Cx43 mutant mouse model of human disease. ONCOGENE. (2011) 30:1681–92. doi: 10.1038/onc.2010.551 21151177

[62] Taylor SC Rosselli-Murai LK Crobeddu B Plante I. A critical path to producing high quality, reproducible data from quantitative western blot experiments. Sci Rep. (2022) 12:17599. doi: 10.1038/s41598-022-22294-x 36266411 PMC9585080

[63] Minn AJ Gupta GP Siegel PM Bos PD Shu W Giri DD. Genes that mediate breast cancer metastasis to lung. Nature. (2005) 436:518–24. doi: 10.1038/nature03799 PMC1283098 16049480

[64] Jin L Han B Siegel E Cui Y Giuliano A Cui X. Breast cancer lung metastasis: Molecular biology and therapeutic implications. Cancer Biol Ther. (2018) 19:858–68. doi: 10.1080/15384047.2018.1456599 PMC6300341 29580128

[65] Gorbokon N Timm P Dum D Menz A Buscheck F Volkel C. Mammaglobin-A expression is highly specific for tumors derived from the breast, the female genital tract, and the salivary gland. Diagnostics (Basel). (2023) 13. doi: 10.3390/diagnostics13061202 PMC10047670 36980510

[66] Kerdelhue B Forest C Coumoul X. Dimethyl-Benz(a)anthracene: A mammary carcinogen and a neuroendocrine disruptor. Biochim Open. (2016) 3:49–55. doi: 10.1016/j.biopen.2016.09.003 29450131 PMC5801823

[67] Jordan VC. Laboratory models of breast cancer to aid the elucidation of antiestrogen action. J Lab Clin Med. (1987) 109:267–77. 3102656

[68] Russo J Gusterson BA Rogers AE Russo IH Wellings SR van Zwieten MJ. Comparative study of human and rat mammary tumorigenesis. Lab Invest. (1990) 62:244–78. 2107367

[69] Abba MC Zhong Y Lee J Kil H Lu Y Takata Y. DMBA induced mouse mammary tumors display high incidence of activating Pik3caH1047 and loss of function Pten mutations. Oncotarget. (2016) 7:64289–99. doi: 10.18632/oncotarget.11733 PMC5325442 27588403

[70] Padovani M Cheng R. Gene expression profiling of mammary glands at an early stage of DMBA-induced carcinogenesis in the female Sprague-Dawley rat. Eur J Oncol = Giornale europeo di oncologia. (2016) 21:21–37. 36213255 PMC9543070

[71] Nascimento R Otoni K. Histological and molecular classification of breast cancer: what do we know? Mastology. (2020) 30. doi: 10.29289/Z25945394

[72] Orrantia-Borunda E Anchondo-Nunez P Acuna-Aguilar LE Gomez-Valles FO Ramirez-Valdespino CA. Subtypes of breast cancer. In: Mayrovitz HN, editor. Breast Cancer. Brisbane (AU): Exon Publications (2022). doi: 10.36255/exon-publications-breast-cancer-subtypes 36122153

[73] Perou CM Sorlie T Eisen MB van de Rijn M Jeffrey SS Rees CA. Molecular portraits of human breast tumours. Nature. (2000) 406:747–52. doi: 10.1038/35021093 10963602

[74] Bertucci F Finetti P Cervera N Esterni B Hermitte F Viens P. How basal are triple-negative breast cancers? Int J Cancer. (2008) 123:236–40. doi: 10.1002/ijc.23518 18398844

[75] Krishnamurti U Silverman JF. HER2 in breast cancer: a review and update. Adv Anat Pathol. (2014) 21:100–7. doi: 10.1097/PAP.0000000000000015 24508693

[76] Hon JD Singh B Sahin A Du G Wang J Wang VY. Breast cancer molecular subtypes: from TNBC to QNBC. Am J Cancer Res. (2016) 6:1864–72. PMC5043099 27725895

[77] Barnard ME Boeke CE Tamimi RM. Established breast cancer risk factors and risk of intrinsic tumor subtypes. Biochim Biophys Acta. (2015) 1856:73–85. doi: 10.1016/j.bbcan.2015.06.002 26071880

[78] Miller JL Bartlett AP Harman RM Majhi PD Jerry DJ Van de Walle GR. Induced mammary cancer in rat models: pathogenesis, genetics, and relevance to female breast cancer. J Mammary Gland Biol Neoplasia. (2022) 27:185–210. doi: 10.1007/s10911-022-09522-w 35904679

[79] Lin Y Yao Y Liu S Wang L Moorthy B Xiong D. Role of mammary epithelial and stromal P450 enzymes in the clearance and metabolic activation of 7,12-dimethylbenz(a)anthracene in mice. Toxicol Lett. (2012) 212:97–105. doi: 10.1016/j.toxlet.2012.05.005 22595614 PMC3668431

[80] Trombino AF Near RI Matulka RA Yang S Hafer LJ Toselli PA. Expression of the aryl hydrocarbon receptor/transcription factor (AhR) and AhR-regulated CYP1. Breast Cancer Res Treat. (2000) 63:117–31. doi: 10.1023/A:1006443104670 11097088

[81] Currier N Solomon SE Demicco EG Chang DL Farago M Ying H. Oncogenic signaling pathways activated in DMBA-induced mouse mammary tumors. Toxicol Pathol. (2005) 33:726–37. doi: 10.1080/01926230500352226 16263698

[82] Marxfeld H Grenet O Bringel J Staedtler F Harleman JH. Differentiation of spontaneous and induced mammary adenocarcinomas of the rat by gene expression profiling. Exp Toxicol Pathol. (2006) 58:151–61. doi: 10.1016/j.etp.2006.06.008 16905300

[83] Arif JM Smith WA Gupta RC. Tissue distribution of DNA adducts in rats treated by intramammillary injection with dibenzo[a,l]pyrene, 7,12-dimethylbenz[a]anthracene and benzo[a]pyrene. Mutat Res. (1997) 378:31–9. doi: 10.1016/S0027-5107(97)00095-X 9288883

[84] Russo J Tay LK Russo IH. Differentiation of the mammary gland and susceptibility to carcinogenesis. Breast Cancer Res Treat. (1982) 2:5–73. doi: 10.1007/BF01805718 6216933

[85] Russo J Tait L Russo IH. Susceptibility of the mammary gland to carcinogenesis. III. The cell of origin of rat mammary carcinoma. Am J Pathol. (1983) 113:50–66. 6312803 PMC1916301

[86] N'Jai AU Larsen M Shi L Jefcoate CR Czuprynski CJ. Bone marrow lymphoid and myeloid progenitor cells are suppressed in 7,12-dimethylbenz(a)anthracene (DMBA) treated mice. Toxicology. (2010) 271:27–35. doi: 10.1016/j.tox.2010.02.009 20171256 PMC2854224

[87] OEHHA. (2017). Evidence on the Carcinogenicity of Coumarin. https://oehha.ca.gov/media/downloads/crnr/coumarinhid.pdf (Accessed August 18, 217)

[88] Feuer G Kellen JA Kovacs K. Suppression of 7,12-dimethylbenz(alpha) anthracene-induced breast carcinoma by coumarin in the rat. Oncology. (1976) 33:35–9. doi: 10.1159/000225098 824591

[89] Wattenberg LW Lam LK Fladmoe AV. Inhibition of chemical carcinogen-induced neoplasia by coumarins and alpha-angelicalactone. Cancer Res. (1979) 39:1651–4. 106961

[90] Holcomb M Safe S. Inhibition of 7,12-dimethylbenzanthracene-induced rat mammary tumor growth by 2,3,7,8-tetrachlorodibenzo-p-dioxin. Cancer Lett. (1994) 82:43–7. doi: 10.1016/0304-3835(94)90144-9 8033067

[91] Brown DJ Van Overmeire I Goeyens L Denison MS De Vito MJ Clark GC. Analysis of Ah receptor pathway activation by brominated flame retardants. Chemosphere. (2004) 55:1509–18. doi: 10.1016/j.chemosphere.2003.10.019 15099731

[92] Anderson LE Morris JE Sasser LB Stevens RG. Effect of constant light on DMBA mammary tumorigenesis in rats. Cancer Lett. (2000) 148:121–6. doi: 10.1016/S0304-3835(99)00320-1 PMC7127503 10695987

[93] Liu Y Yin T Feng Y Cona MM Huang G Liu J. Mammalian models of chemically induced primary Malignancies exploitable for imaging-based preclinical theragnostic research. Quant Imaging Med Surg. (2015) 5:708–29. doi: 10.3978/j.issn.2223-4292.2015.06.01 PMC4671963 26682141

[94] Betancourt AM Eltoum IA Desmond RA Russo J Lamartiniere CA. *In utero* exposure to bisphenol A shifts the window of susceptibility for mammary carcinogenesis in the rat. Environ Health Perspect. (2010) 118:1614–9. doi: 10.1289/ehp.1002148 PMC2974702 20675265

[95] Moral R Wang R Russo IH Lamartiniere CA Pereira J Russo J. Effect of prenatal exposure to the endocrine disruptor bisphenol A on mammary gland morphology and gene expression signature. J Endocrinol. (2008) 196:101–12. doi: 10.1677/JOE-07-0056 18180321

[96] Russo J Russo IH. DNA labeling index and structure of the rat mammary gland as determinants of its susceptibility to carcinogenesis. J Natl Cancer Inst. (1978) 61:1451–9. 102857

[97] Hurley S Goldberg D Park JS Petreas M Bernstein L Anton-Culver H. A breast cancer case-control study of polybrominated diphenyl ether (PBDE) serum levels among California women. Environ Int. (2019) 127:412–9. doi: 10.1016/j.envint.2019.03.043 PMC6522143 30954728

[98] Darnerud PO. Toxic effects of brominated flame retardants in man and in wildlife. Environ Int. (2003) 29:841–53. doi: 10.1016/S0160-4120(03)00107-7 12850100

[99] Hardell L Bavel B Lindström G Eriksson M Carlberg M. *In utero* exposure to persistent organic pollutants in relation to testicular cancer risk. Int J Androl. (2006) 29:228–34. doi: 10.1111/j.1365-2605.2005.00622.x 16371110

[100] Ward MH Colt JS Deziel NC Whitehead TP Reynolds P Gunier RB. Residential levels of polybrominated diphenyl ethers and risk of childhood acute lymphoblastic leukemia in California. Environ Health Perspect. (2014) 122:1110–6. doi: 10.1289/ehp.1307602 PMC4181922 24911217

[101] Mercado-Feliciano M Bigsby RM. Hydroxylated metabolites of the polybrominated diphenyl ether mixture DE-71 are weak estrogen receptor-alpha ligands. Environ Health Perspect. (2008) 116:1315–21. doi: 10.1289/ehp.11343 PMC2569088 18941571

[102] Mercado-Feliciano M Bigsby RM. The polybrominated diphenyl ether mixture DE-71 is mildly estrogenic. Environ Health Perspect. (2008) 116:605–11. doi: 10.1289/ehp.10643 PMC2367668 18470304

[103] EPA. Toxicological review of decabromodiphenyl ether (BDE-209). U.S. Environmental Protection Agency.: U.S. Environmental Protection Agency (2008). Available at: https://cfpub.epa.gov/ncea/iris/search/index.cfm?keyword=BDE-209.

[104] Albano GD Moscato M Montalbano AM Anzalone G Gagliardo R Bonanno A. Can PBDEs affect the pathophysiologic complex of epithelium in lung diseases? Chemosphere. (2020) 241:125087. doi: 10.1016/j.chemosphere.2019.125087 31622892

[105] Koike E Yanagisawa R Takano H. Brominated flame retardants, hexabromocyclododecane and tetrabromobisphenol A, affect proinflammatory protein expression in human bronchial epithelial cells via disruption of intracellular signaling. Toxicol In Vitro. (2016) 32:212–9. doi: 10.1016/j.tiv.2015.12.013 26718265

