# Supplementary figures and images for: Exposure to brominated flame retardants in utero and through lactation delays the development of DMBA-induced mammary cancer: potential effects on subtypes?
Source: Front Endocrinol (Lausanne). 2024 Nov 14;15:1429142. doi: 10.3389/fendo.2024.1429142 (PMC11602300; doi:10.3389/fendo.2024.1429142)

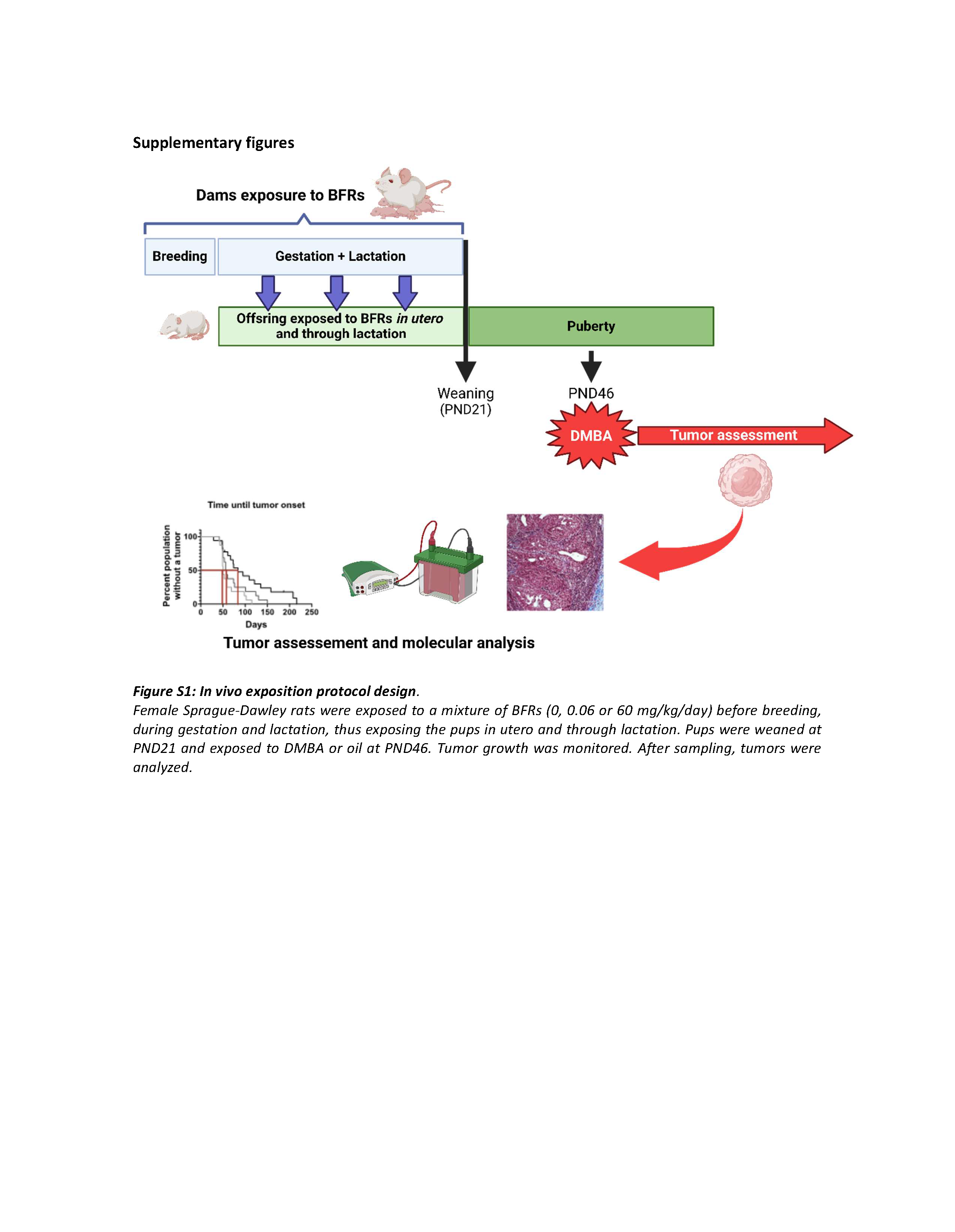

Supplement: Supplementary file 1 [file Image1.tif]

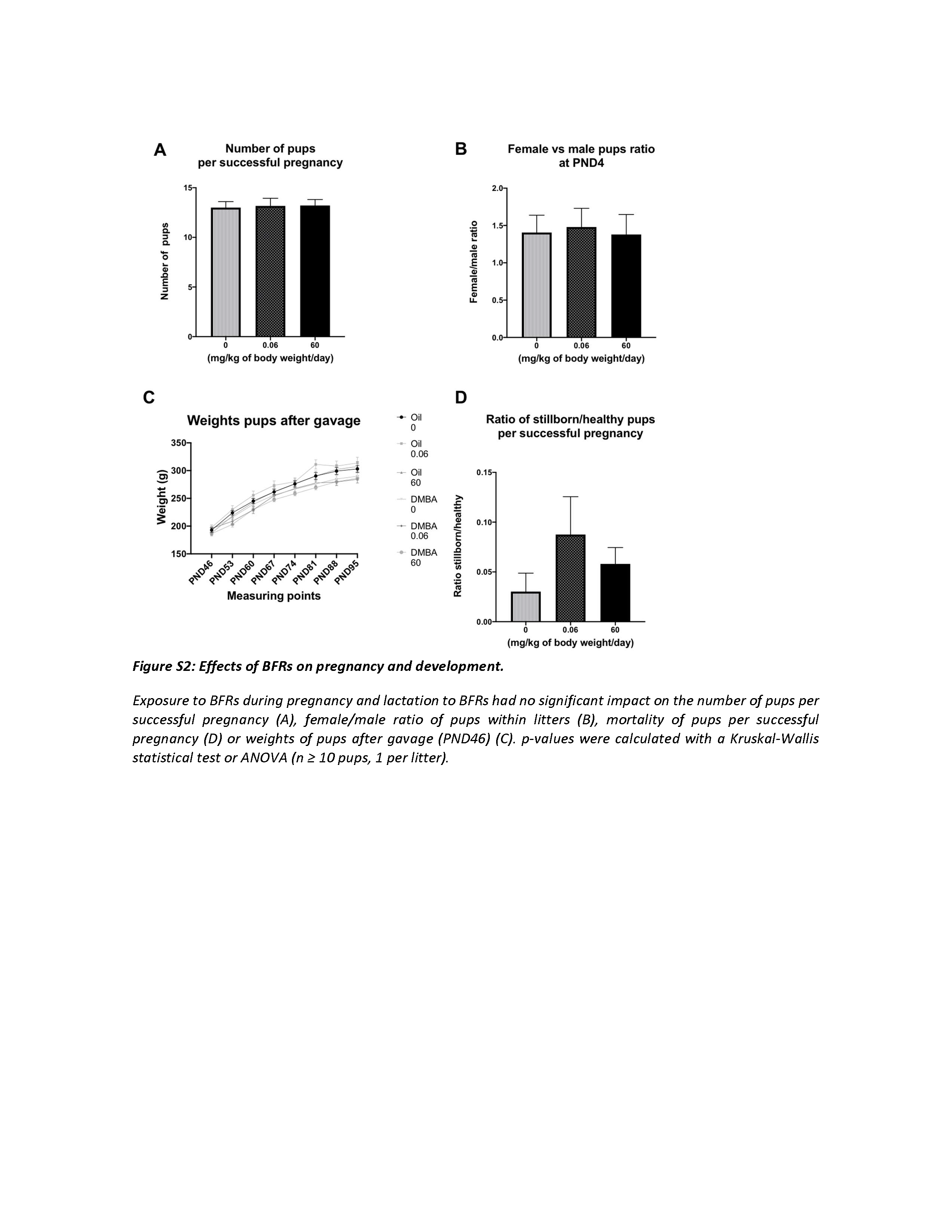

Supplement: Supplementary file 2 [file Image2.tif]

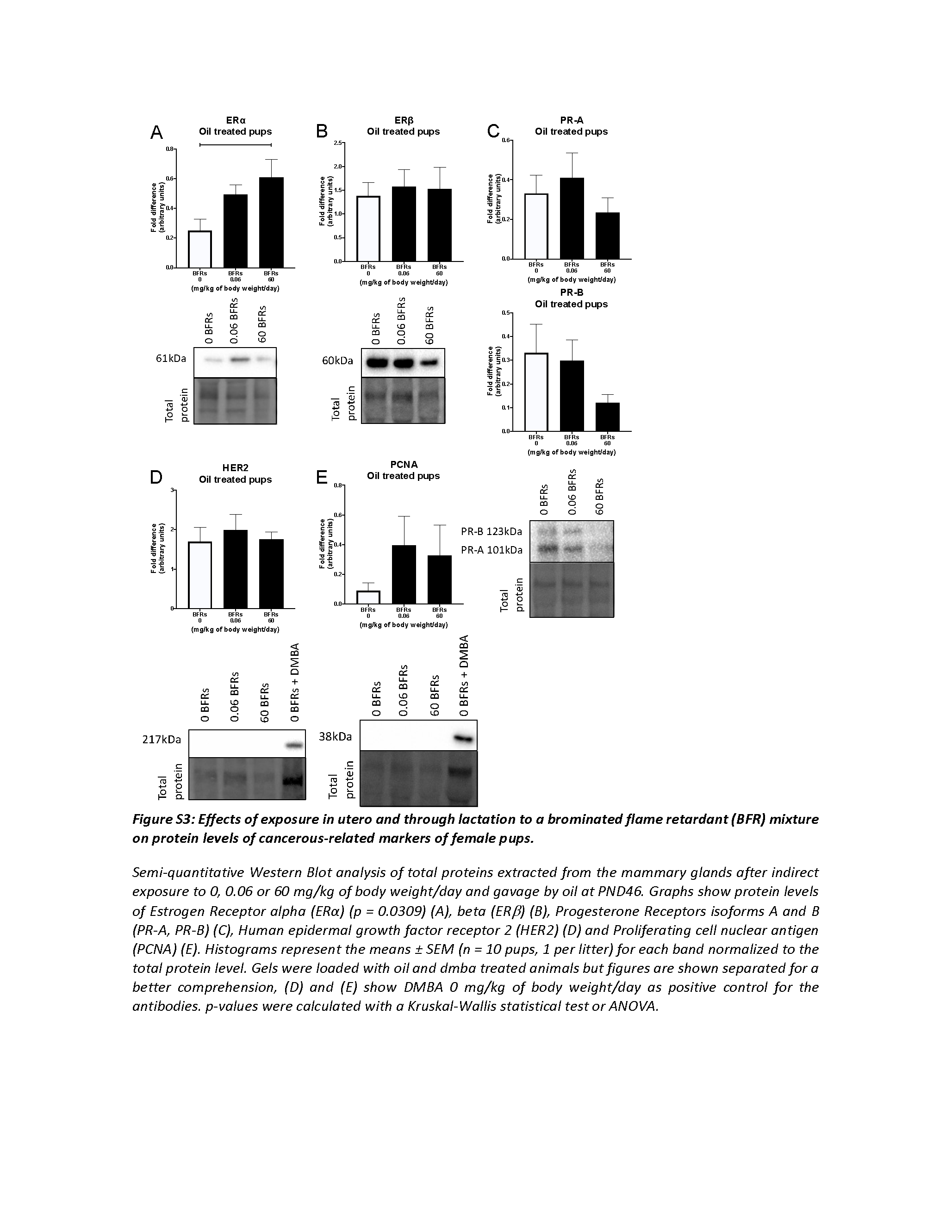

Supplement: Supplementary file 3 [file Image3.tif]

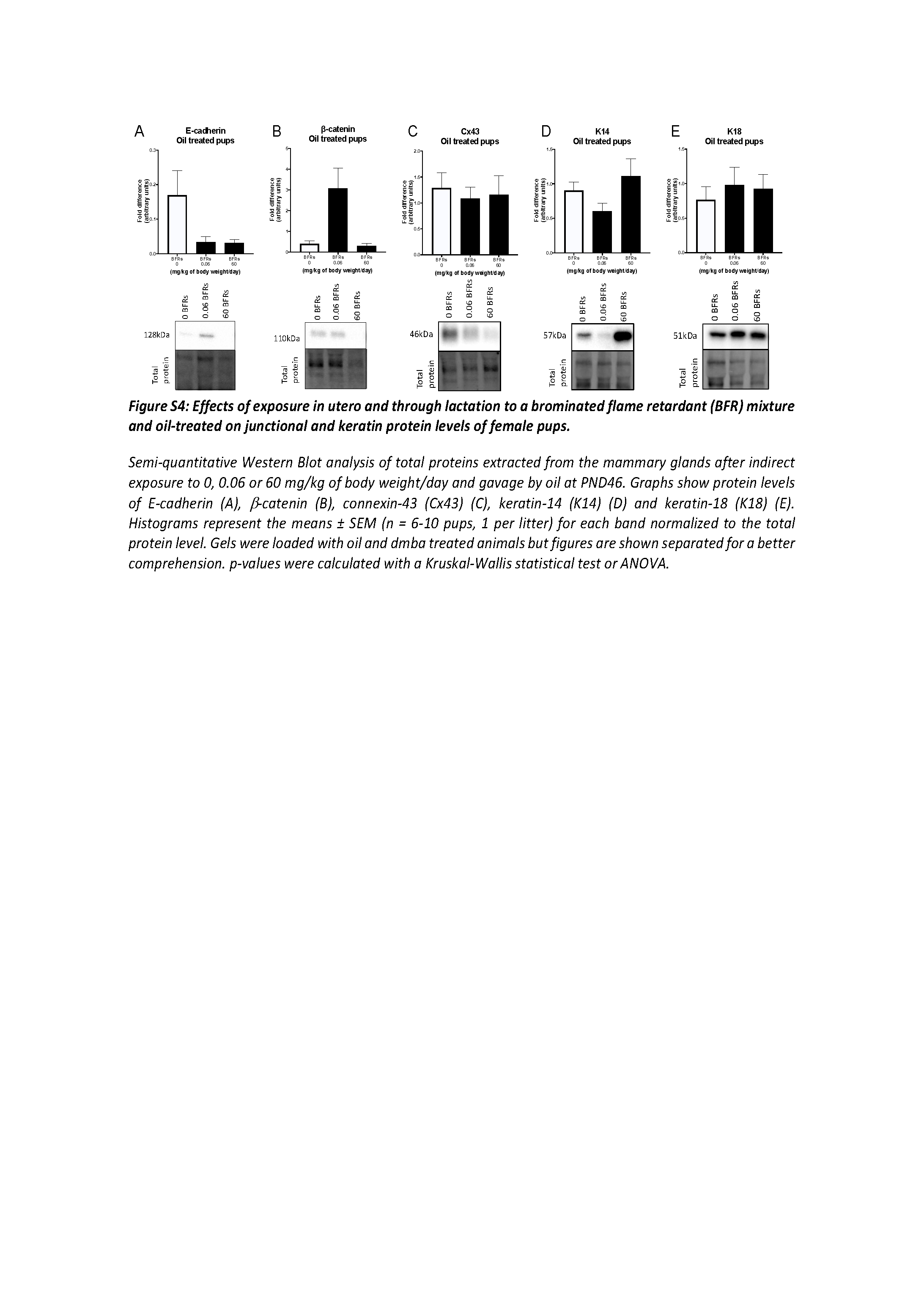

Supplement: Supplementary file 4 [file Image4.tif]

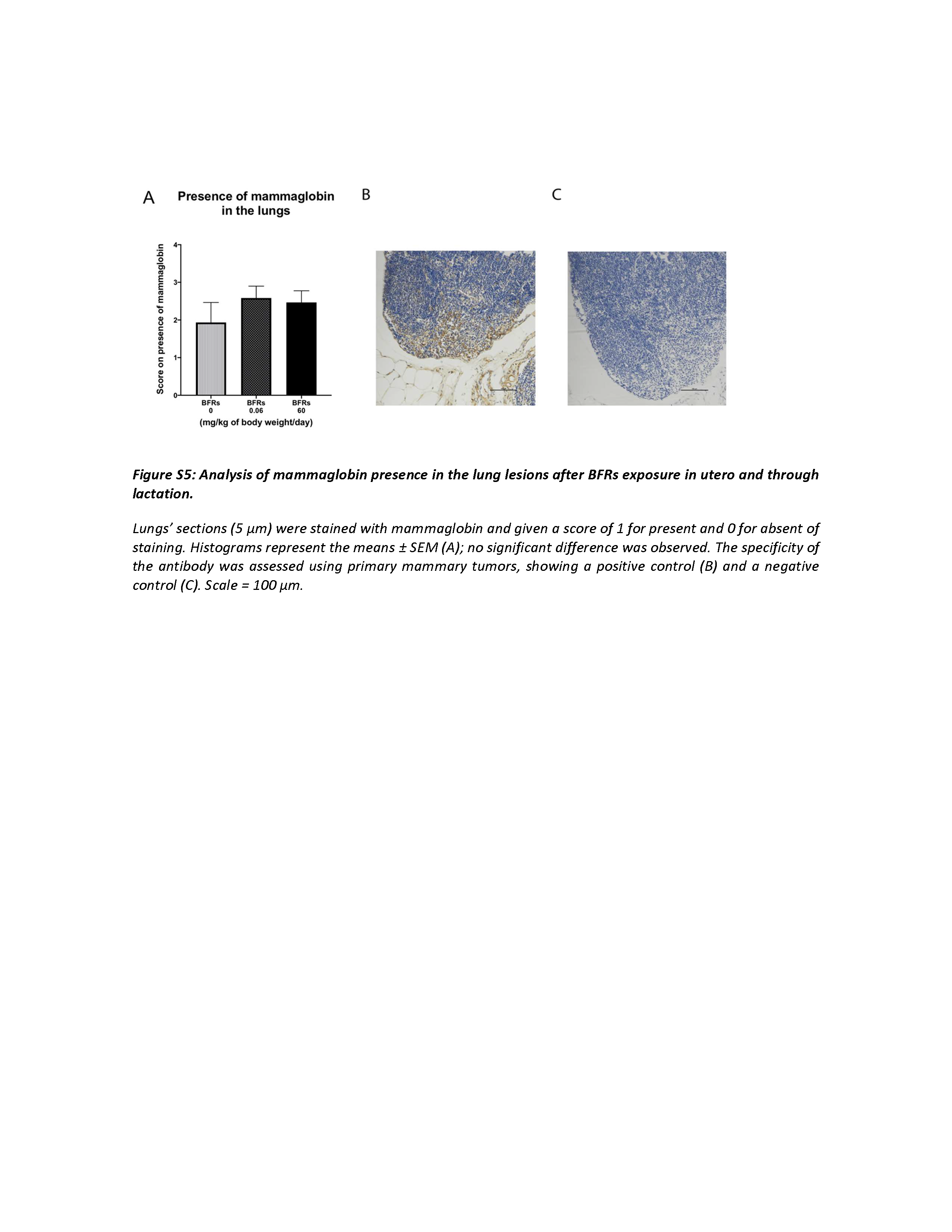

Supplement: Supplementary file 5 [file Image5.tif]

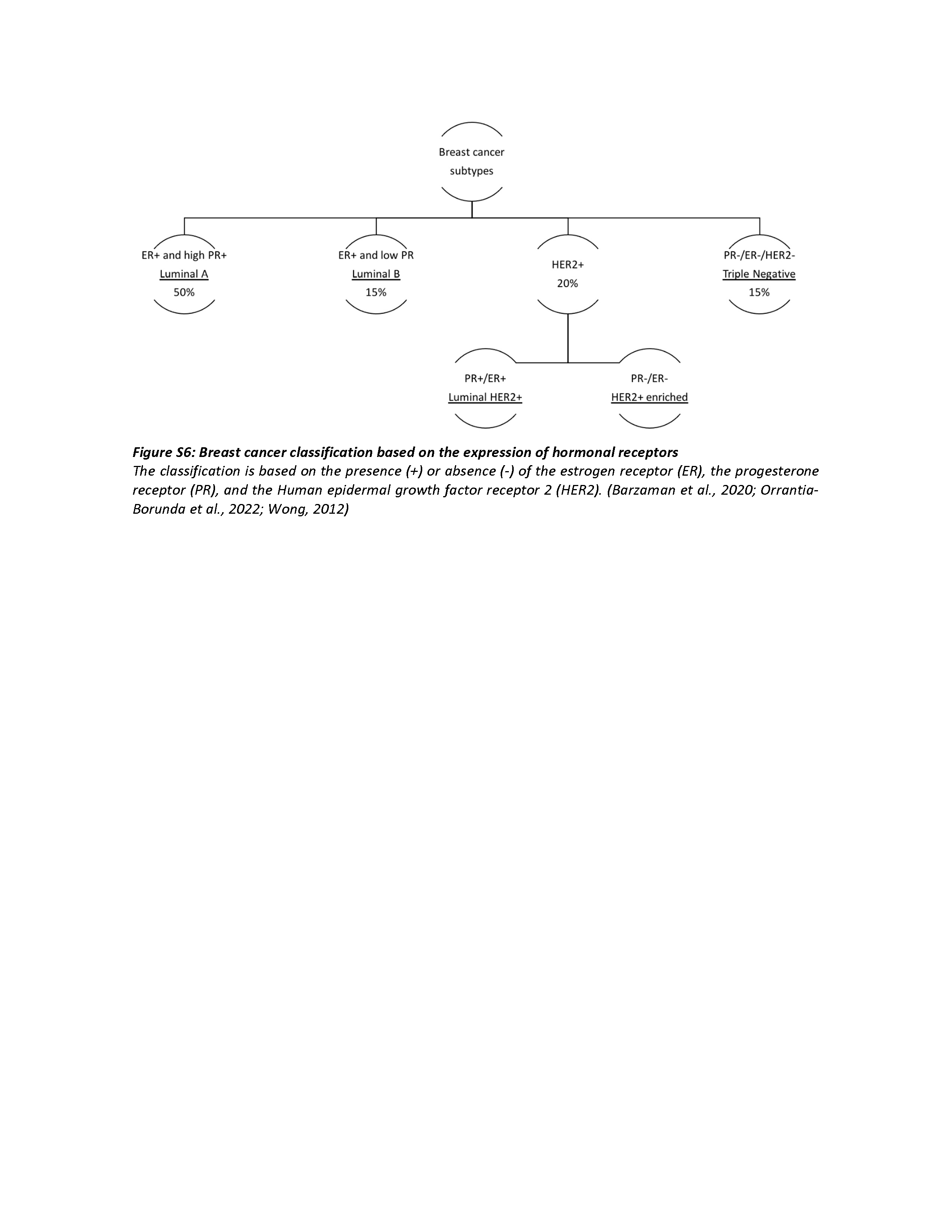

Supplement: Supplementary file 6 [file Image6.tif]
