# Supplementary material for: Exposure to brominated flame retardants in utero and through lactation delays the development of DMBA-induced mammary cancer: potential effects on subtypes?
Source: Front Endocrinol (Lausanne). 2024 Nov 14;15:1429142. doi: 10.3389/fendo.2024.1429142 (PMC11602300; doi:10.3389/fendo.2024.1429142)
Supplement: Supplementary file 7 [file Table1.pdf]

**Supplementary Table 1: Technical PBDE mixtures used for the treatment**

| Chemical | Acronym | CAS # | Source | Purity |
|---|---|---|---|---|
| Pentabromodiphenyl ether mixture | DE-71 | 32534-81-9 | Chemtura (Laurenceville, GA) via Dr. Doug Arnold, Health Canada | Technical Mixture |
| Octabromodiphenyl ether mixture | DE-79 | 32536-52-0 | Chemtura (Laurenceville, GA) via Wellington Laboratories (Guelph, ON) | Technical Mixture |
| Decabromodiphenyl ether | BDE-209 | 1163-19-5 | Matrix scientific | 95% |
| Hexabromocyclododecane | HBCDD | 3194-55-6 | Sigma-Aldrich | 95% |
