# Supplementary material for: Exposure to brominated flame retardants in utero and through lactation delays the development of DMBA-induced mammary cancer: potential effects on subtypes?
Source: Front Endocrinol (Lausanne). 2024 Nov 14;15:1429142. doi: 10.3389/fendo.2024.1429142 (PMC11602300; doi:10.3389/fendo.2024.1429142)
Supplement: Supplementary file 8 [file Table2.pdf]

Supplementary Table 2: Antibodies and conditions used for western blot analysis

Target protein	Host	Dilution	Catalogue number	Manufacturer
**Primary antibodies**				
β-catenin	Rabbit	1/1000	8480	Cell signaling
E-cadherin	Mouse	1/1000	14472	Cell signaling
Cx43	Rabbit	1/500	C6219	Sigma Aldrich
K14	Mouse	1/200	ms-115-p1	Thermofisher
K18	Rabbit	1/1000	ab52948	Abcam
HER2	Rabbit	1/1000	2165	Cell signaling
PCNA	Mouse	1/2000	2586S	Cell signaling
PR A/B	Rabbit	1/500	Ab16661	Abcam
ERα	Rabbit	1/500	75635	Abcam
ERβ	Rabbit	1/1000	PA 1-310B	Thermofisher
**Secondary antibodies**				
anti-mouse IgG HRP-linked antibody	Horse	1/10 000	7076s	Cell signaling
Anti-rabbit IgG HRP-linked antibody	Goat	1/10 000	7074s	Cell signaling
